# Influence of grape consumption on the human microbiome

**DOI:** 10.1038/s41598-023-34813-5

**Published:** 2023-05-12

**Authors:** Asim Dave, Diren Beyoğlu, Eun-Jung Park, Jeffrey R. Idle, John M. Pezzuto

**Affiliations:** 1 Immunology Program, Memorial Sloan Kettering Cancer Center, New York, NY 10065 USA; 2 College of Pharmacy and Health Sciences, Western New England University, 1215 Wilbraham Rd., Springfield, MA 01119 USA; 3 Division of Pharmaceutical Sciences, Arnold & Marie Schwartz College of Pharmacy and Health Sciences, Long Island University, Brooklyn, NY 11201 USA; 4 Department of Medicine, UMass Chan Medical School—Baystate, Springfield, MA 01199 USA

**Keywords:** Metabolomics, Metagenomics

## Abstract

Over the years, a substantial body of information has accumulated suggesting dietary consumption of grapes may have a positive influence on human health. Here, we investigate the potential of grapes to modulate the human microbiome. Microbiome composition as well as urinary and plasma metabolites were sequentially assessed in 29 healthy free-living male (age 24–55 years) and female subjects (age 29–53 years) following two-weeks of a restricted diet (Day 15), two-weeks of a restricted diet with grape consumption (equivalent to three servings per day) (Day 30), and four-weeks of restricted diet without grape consumption (Day 60). Based on alpha-diversity indices, grape consumption did not alter the overall composition of the microbial community, other than with the female subset based on the Chao index. Similarly, based on beta-diversity analyses, the diversity of species was not significantly altered at the three time points of the study. However, following 2 weeks of grape consumption, taxonomic abundance was altered (e.g., decreased *Holdemania* spp. and increased *Streptococcus thermophiles*), as were various enzyme levels and KEGG pathways. Further, taxonomic, enzyme and pathway shifts were observed 30 days following the termination of grape consumption, some of which returned to baseline and some of which suggest a delayed effect of grape consumption. Metabolomic analyses supported the functional significance of these alterations wherein, for example, 2′-deoxyribonic acid, glutaconic acid, and 3-hydroxyphenylacetic acid were elevated following grape consumption and returned to baseline following the washout period. Inter-individual variation was observed and exemplified by analysis of a subgroup of the study population showing unique patterns of taxonomic distribution over the study period. The biological ramifications of these dynamics remain to be defined. However, while it seems clear that grape consumption does not perturb the eubiotic state of the microbiome with normal, healthy human subjects, it is likely that shifts in the intricate interactive networks that result from grape consumption have physiological significance of relevance to grape action.

## Introduction

The potential influence of the human microbiome, consisting of over 3 million genes and on the order of 10^14^ microorganisms^1^, on health and well-being is profound. Over the past two decades, remarkable strides in microbiome research have provided the tools and knowledge to allow meaningful investigation of the influence of this “tissue” on human health and disease (cf.^2^). Words such as prebiotic, probiotic, synbiotic, eubiosis and dysbiosis are now commonly incorporated in the ordinary lexicon of the lay public and scientific community. The marketplace has expanded into a multi-billion dollar industry, with substantial growth anticipated in the future, through provision of products designed for humans as well as other mammals.

Given it is generally agreed that maintaining a “healthy” gut microbiome is important for human health, the potential impact of diet has been widely studied^3^. As such, modulation of microbiome composition as well as the levels of metabolites generated by the microbiome (such as acetate, propionate and butyrate), by dietary consumption of protein, carbohydrate, fat, polyphenols, phytoestrogens, etc., have been described^4^.

An area of interest for us is the potential influence of grapes on health. Dietary consumption is prevalent, as reflected by the production of over 6 million tons per year in the US alone. Based on human clinical trials, or studies conducted with animal models, results have suggested an array of responses mediated by the grape on atherosclerosis, inflammation, cancer, gastrointestinal health, CNS effects, osteoarthritis, urinary bladder function, and vision^5^.

Recently, employing mouse models provided with dietary grapes, we have shown remarkable effects on gene expression, prevention or delay of fatty liver, and enhancement of lifespan^6^, as well as effects on cognition and gene expression in the brain^7^. Although a prominent chemical constituent of the grape is resveratrol, the biologic potential of which has been extensively investigated^8^, only small quantities of this substance are provided through a normal human diet. Moreover, the grape is known to contain over 1600 phytochemical constituents^9^, many of which either alone or in combination may be capable of mediating a response. As such, in the context of diet and health, it is of greatest relevance to consider the grape as a whole-food.

Considering the broad scope of actions associated with grape consumption, we set out to explore the potential influence on the human microbiome as a possible mechanistic underpinning. In previous work, treatment of the human intestinal microbiota with total grape seed polyphenols led to a shift in the profiles of short-chain fatty acids (SCFAs) and relevant microbial populations^10^. With mice given a high-fat diet supplemented with enriched grape powder, the abundance of genes that regulate microbial production of butyrate were selectively increased^11^. In our murine work, urinary excretion of the gut microbiota metabolites 4-hydroxyphenylacetic acid, 5-hydroxyindole, glyceric acid, gluconic acid and *myo*-inositol was attenuated when grape was added to a standard diet, and the gut microbiota metabolites gluconic acid, *scyllo*-inositol, mannitol, xylitol, 5-hydroxyindole and 2′-deoxyribonic acid were increased in urine when grape was added to a high-fat diet^12–14^. Further, in a two-phase human intervention study (4-week standardization phase and a 4-week grape intervention phase), Yang et al. observed an increase in the alpha-diversity index of the gut microbiome (Shannon index), as well as a decrease in total cholesterol and total bile acid^15^.

Using an alternate protocol and a larger number of subjects, we currently report a human trial in which normal free-living volunteers consumed the equivalent of three servings of grapes per day for two weeks, followed by a washout period of one month. The resulting composition of the gut microbiome was assessed through fecal analysis. In addition, an evaluation of metabolites in urine and plasma was performed.

## Results

### Grape intervention study design

The trial was conducted over a period of two months. Forty normal, healthy, free-living human subjects entered the trial in which plasma, urine and fecal material were sequentially collected following two-weeks of restricted diet (Day 15), two-weeks of restricted diet supplemented with the equivalent of three servings of grapes per day (Day 30), and a one month washout period (Day 60). No adverse events occurred during the entire study period. Twenty-nine subjects completed all phases of the trial.

### Effect of dietary grape consumption on gut microbiota

#### Alpha-diversity

Alpha-diversity was investigated to assess the richness (number) or evenness (relative abundance) of the microbiome of the study population on Days 15, 30 and 60 (Supplement 1). With all 29 subjects taken together for the analysis, there was no difference found in the OTUs, Chao1 or Shannon diversity indices. Similarly, no alterations were observed with males included in the study group, ages 24–44. However, with females in the study group, 29–39 years of age, there was a difference observed at Day 30 (following two weeks of grape consumption) where Cohen's *d* effect size for the Chao test was 0.836 and the *p* value was 0.114 (Student’s paired *t*-test). A difference was also observed when comparing the baseline (Day 15) with the washout period (60th day) (Cohen's *d* of 0.982 and a *P* value of 0.079).

#### Beta-diversity

Beta-diversity assessed by Bray–Curtis dissimilarity was calculated and visualized via principal component analyses (PCA) and principal coordinate analyses (PCoA) (Supplement 2). According to cluster analyses (95% confidence intervals), no significant differences were observed on Days 15, 30 or 60 when the study population was evaluated as a whole or divided into groups comprised of males only or females only.

#### Sequencing of the 16S rDNA reveals alterations in microbial content

**Figure 1:**
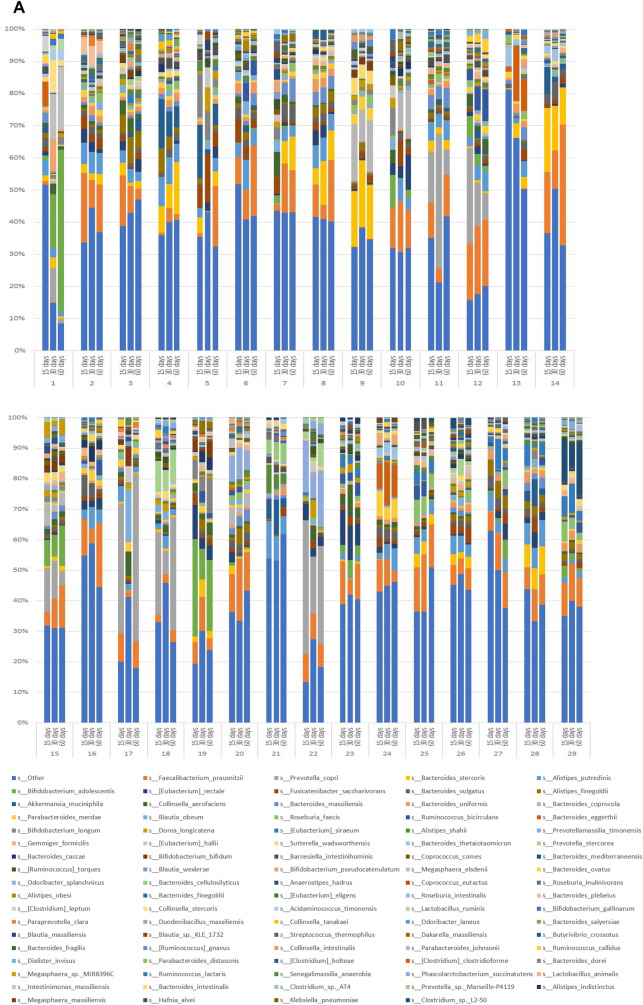

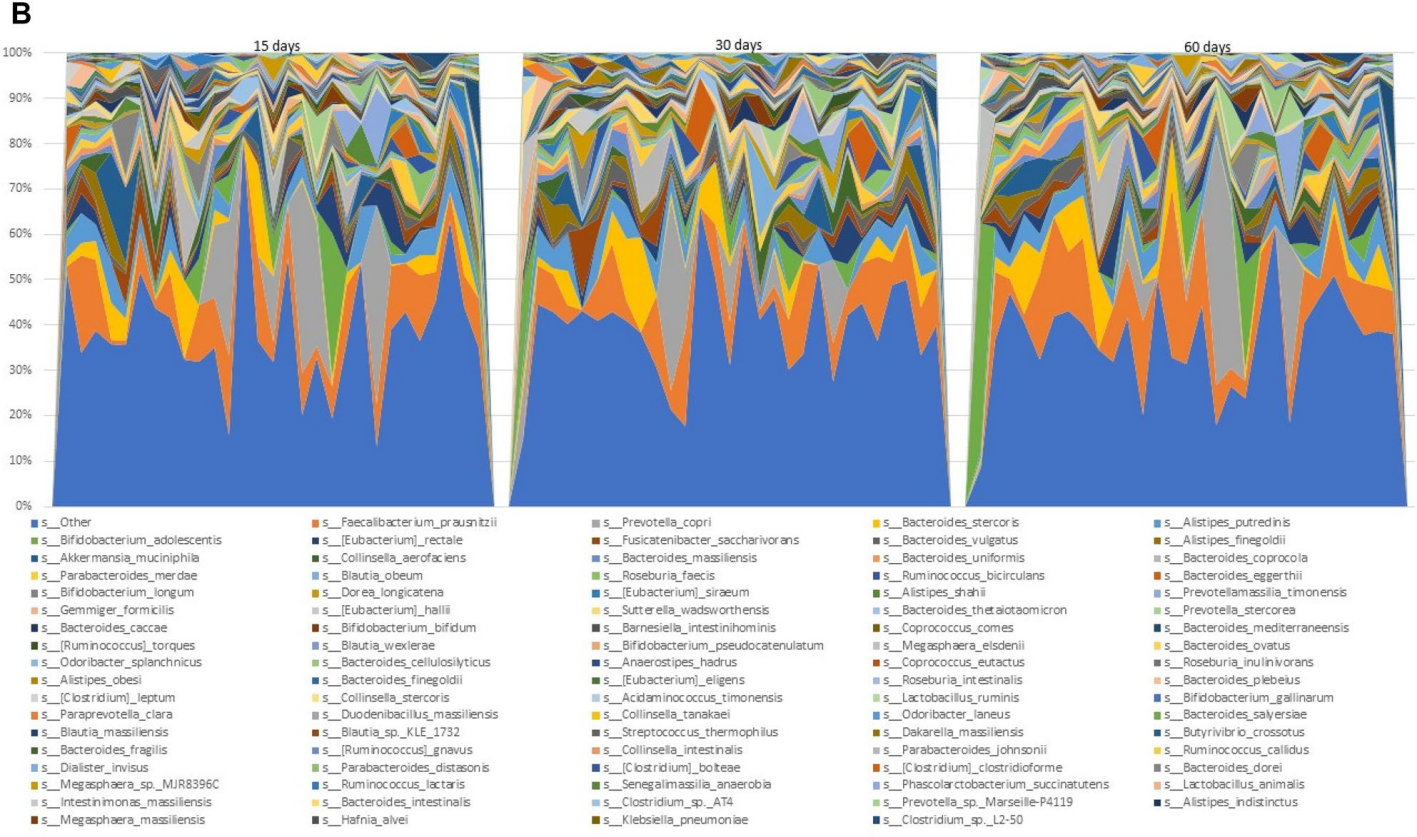
Charts representing diversity of the species. (**A**) Stacked plots present the diversity of all the species in individual subjects from 1 to 29. (**B**) Area charts show the diversity of species among the 29 subjects on Day 15, 30 and 60.

Microbial species found in specimens from all 29 subjects on Day 15, 30 and 60 are shown in Fig. 1A. The most abundant species found in the gut microbiota include *Faecalibacterium prausnitzii*, *Prevotella copri*, *Bacteroides stercoris*, *Alistipes putredinis*, *Bifidobacterium adolescentis*, *Eubacterium rectale*, *Fusicatenibacter saccharivorans*, *Bacteroides vulgatus*, *Alistipes finegoldii*, *Akkermansia muciniphila*, *Collinsella aerofaciens*, *Bacteroides uniformis*, *Bacteroides coprocola* and *Parabacteroides merdae*. As can be gleaned by comparisons of the triplet bars in Fig. 1A, each time point with each individual shows a different profile suggestive of change being induced by the dietary protocol. An overview of the individual variation of diversity of all the species on Days 15, 30 and 60 days can be visualize by the area charts shown in Fig. 1B, and transitions in microbial composition from Day 15 to 30, Day 15 to 60, and Day 15 to 60, are evident from the overlays of the area charts shown in Supplement 3, Fig. S1.

### Analysis of taxonomic abundance for all subjects

Comparative microbial taxonomic analyses for the entire subject population for Day 15 vs. 30, Day 30 vs. 60, and Day 15 vs. 60 are presented in Tables 1, 2, 3, respectively. Selections included in the Tables were deemed to be those influenced most greatly by dietary status based on strong *p*-values as well as Cohen *d-*values indicating an effect size in the medium to large range. Taxonomic hierarchy is designated as well as brief functional connotation for each entry.

**Table 1:** Comparison of the taxonomy of Day 15 vs. 30 days in all subjects taken together.

Taxonomy	Log2 (Fold-change)	*P* value	Cohen's *D*	Functional connotations
g__*Holdemania*^a^	− 1.902	0.004	0.763	Reduced in vegetarian diet^16^
s__*Eubacterium eligens*	− 1.465	0.013	0.667	Bile acid and cholesterol transformations in the gut, thereby^17^ contributing to their homeostasis
c__Erysipelotrichia	− 0.590	0.078	0.485	Found to be enriched in colorectal cancer^18^
o__Erysipelotrichales	− 0.590	0.078	0.485	Found to be enriched in colorectal cancer^18^
f__*Erysipelotrichaceae*	− 0.590	0.078	0.485	Found to be enriched in colorectal cancer^17^
s__*Clostridium clostridioforme*^b^	0.697	0.081	0.492	Associated with serious or invasive human infections^19^ prior to reclassification^b^
s__*Streptococcus thermophilus*	2.350	0.090	0.434	*Streptococcus thermophilus* strains associated with the assumption of health benefits of yogurt consumption^20^

^a^Taxonomic hierarchies are designated as c (class), o (order), f (family), g (genus) or s (species).

^b^*Clostridium clostridioforme* has been reclassified as *Enterocloster clostridioformis* (*Lachnospiraceae*), the latter of which is a benign member of gut microbiomes known for plant-degrading potential^21^.

**Table 2:** Comparison of the taxonomy of Day 30 vs. 60 in all subjects taken together.

Taxonomy	Log2 (Fold-change)	*P* value	Cohen's *D*	Functional connotations
g__*Holdemania*^a^	1.822	0.005	0.743	Reduced in vegetarian diet^16^
s__*Blautia wexlerae*	0.557	0.032	0.602	Depletion is associated with insulin resistance in obese individuals^22^
g__*Clostridium*	− 0.636	0.042	0.536	Described as leading species in the maintenance of gut homeostasis^21^ (see footnote b, Table 1)
o__Propionibacteriales	2.955	0.053	0.451	Produce microbial metabolites such as short-chain fatty acids during glucose fermentation^23^
f__*Propionibacteriaceae*	2.955	0.053	0.451	Produce microbial metabolites such as short-chain fatty acids during glucose fermentation^23^
c__Coriobacteriia	0.919	0.066	0.458	Bile acid metabolism^24^
g__*Blautia*	0.661	0.070	0.487	Depletion in the gut ecosystem may occur in cases of obesity and contribute to metabolic inflammation leading to insulin resistance^22^
f__*Coriobacteriaceae*	0.992	0.072	0.445	Bile acid metabolism^24^
s__*Ruminococcus torques*	1.017	0.074	0.447	Decrease gut barrier integrity, and a decrease in *Lactobacillus johnsonii*, a bacterium that helps maintain the intestinal epithelial cell layer^25^
o__Coriobacteriales	0.973	0.077	0.437	Bile acid metabolism^24^

^a^Taxonomic hierarchies are designated as c (class), o (order), f (family), g (genus) or s (species).

**Table 3:** Comparison of the taxonomy of Day 15 vs. 60 in all subjects taken together.

Taxanomy	Log2 (Fold-change)	*P* value	Cohen's *D*	Functional connotations
f__*Christensenellaceae*^a^	1.837	0.055	0.514	Plays an important role in maintaining microbial symbiosis^26^
g__*Christensenella*	1.837	0.055	0.514	Plays an important role in maintaining microbial symbiosis^26^
g__*Anaerostipes*	1.123	0.059	0.446	Butyrate production^27^
s__*Coprococcus comes*	0.910	0.059	0.452	Known to use a butyrate kinase to produce butyrate^28^
s__*Blautia wexlerae*	0.647	0.052	0.436	Depletion is associated with insulin resistance in obese individuals^22^
s__*Anaerostipes hadrus*	1.199	0.053	0.459	Positive impact on gastrointestinal tract homeostasis by increasing intestinal epithelial cells, expression of tight junction proteins and acts as an anti-inflammatory agent^27^

^a^Taxonomic hierarchies are designated as f (family), g (genus) or s (species).

For each comparison, significant alterations in the microbiome were observed. Notably, following grape consumption (Day 30), *Streptococcus thermophiles*, considered a probiotic that produces lactic acid in the gut, was elevated. On the other hand, *Holdemania* spp., were reduced, as occurs in those on a vegetarian diet. Interestingly, 30 days following grape consumption, *Holdemania* abundance increased, and no change was noted in *Streptococcus thermophiles*. In comparing the 30 day time point to the 60 day time point, consistent increases in abundance of organisms associated with production of metabolites such as short-chain fatty acids were noted. This tends to suggest a delayed response to grape consumption, since these shifts were not observed in the Day 15 vs. 30 or Day 15 vs. 60 comparisons.

### Analysis of enzymic abundance for all subjects

As above, a comparison of enzymic abundance associated with the microbiome was performed for Day 15 vs 30, Day 30 vs 60, and Day 15 vs 60. The results are shown in Tables 4, 5 and 6, respectively. Again, entries were selected on the basis of strong *p*-values as well as Cohen *d-*values indicating an effect size in the medium to large range.

**Table 4:** Enzymes enriched when comparing Day 15 vs. 30 for all subjects.

Enzymes	Log2 (Fold-change)	*P* value	Cohen's *D*
2.3.1.81 aacC; aminoglycoside 3-*N*-acetyltransferase	− 0.804	0.007	0.647
4.1.1.2 oxdD; oxalate decarboxylase	− 1.874	0.009	0.748
1.13.11.2 catE; catechol 2,3-dioxygenase	2.280	0.045	0.509
2.8.3.17 fldA; cinnamoyl-CoA:phenyllactate CoA-transferase	0.666	0.080	0.474
3.1.2.30 mcl2; (3*S*)-malyl-CoA thioesterase	− 2.505	0.081	0.385
3.5.2.6 blaTEM; beta-lactamase class A TEM	1.585	0.082	0.441

**Table 5:** Enzymes enriched when comparing Day 30 vs. 60 for all subjects.

Enzymes	Log2 (Fold-change)	*P* value	Cohen's *D*
1.17.1.10 fdhB; formate dehydrogenase (NADP^+^) beta subunit	0.469	0.016	0.608
1.1.1.301 apdH, APDH; d-arabitol-phosphate dehydrogenase	0.685	0.031	0.595
1.20.4.1 ARSC1, arsC; arsenate reductase	0.411	0.039	0.574
2.7.2.2 arcC; carbamate kinase	0.525	0.041	0.544
2.7.7.7 dnaE2; error-prone DNA polymerase	3.548	0.044	0.558
2.7.1.156 cobP, cobU; adenosylcobinamide kinase/adenosylcobinamide-phosphate guanylyltransferase	0.407	0.049	0.538
2.7.7.62 cobP, cobU; adenosylcobinamide kinase/adenosylcobinamide-phosphate guanylyltransferase	0.407	0.049	0.538
1.17.1.10 fdhA; formate dehydrogenase (NADP^+^) alpha subunit	1.175	0.050	0.504
1.3.1.1 preT; dihydropyrimidine dehydrogenase (NAD^+^) subunit PreT	0.484	0.054	0.545
5.2.1.8 slyD; FKBP-type peptidyl-prolyl *cis–trans* isomerase SlyD	0.281	0.054	0.488
3.1.1.41 cah; cephalosporin-C deacetylase	0.655	0.058	0.433
1.1.1.304 butA, budC; *meso*-butanediol dehydrogenase/(*S,S*)-butanediol dehydrogenase/diacetyl reductase	− 1.357	0.060	0.497
1.1.1.76 butA, budC; *meso*-butanediol dehydrogenase/(*S,S*)-butanediol dehydrogenase/diacetyl reductase	− 1.357	0.060	0.497
4.3.2.6 btrG; gamma-l-glutamyl-butirosin B gamma-l-glutamyl cyclotransferase	2.041	0.061	0.468
3.6.3.40 tagH; teichoic acid transport system ATP-binding protein	0.357	0.062	0.547
4.6.1.1 E4.6.1.1; adenylate cyclase	1.604	0.065	0.448

**Table 6:** Enzymes enriched when comparing Day 15 vs. 60 for all subjects.

Enzymes	Log2 (Fold-change)	*P* value	Cohen's *D*
2.7.7.7 dnaE2; error-prone DNA polymerase	3.812	0.002	0.822
4.1.2.40 gatY-kbaY; tagatose 1,6-diphosphate aldolase GatY/KbaY	1.024	0.017	0.582
1.1.1.14 SORD, gutB; l-iditol 2-dehydrogenase	0.682	0.018	0.588
4.1.1.95 btrK; l-glutamyl-[BtrI acyl-carrier protein] decarboxylase	0.603	0.029	0.527
2.1.1.307 elmMIII; 8-demethyl-8-(2,3-dimethoxy-alpha-l-rhamnosyl)tetracenomycin-C 4′-*O*-methyltransferase	0.581	0.040	0.459
3.1.1.41 cah; cephalosporin-C deacetylase	0.661	0.043	0.524
2.7.1.200 PTS-Gat-EIIB, gatB, sgcB; PTS system, galactitol-specific IIB component	1.074	0.045	0.558
1.3.7.8 bcrB, badE; benzoyl-CoA reductase subunit B	1.085	0.047	0.479
2.7.7.39 tagD; glycerol-3-phosphate cytidylyltransferase	0.566	0.047	0.455
3.5.1.49 E3.5.1.49; formamidase	1.182	0.052	0.378
5.3.3.14 fabM; *trans*-2-decenoyl-[acyl-carrier protein] isomerase	1.032	0.054	0.489
1.17.1.5 ndhF; nicotinate dehydrogenase FAD-subunit	0.470	0.054	0.431
4.1.1.2 oxdD; oxalate decarboxylase	− 1.328	0.063	0.491
1.3.8.13 caiA; crotonobetainyl-CoA dehydrogenase	0.640	0.067	0.446
2.7.8.12 tagF; CDP-glycerol glycerophosphotransferase	0.606	0.067	0.430

The least number of entries is found in Table 4, when comparing Day 15 with Day 30. Notably, however, catechol 2,3-dioxygenase, which may be viewed as having value in metabolic detoxification^29^, was elevated. On the other hand, (3*S*)-malyl-CoA thioesterase was reduced, which may have an effect on glyoxylate cycle of microorganisms.

In comparing Day 15 with Day 30 and 60, a notable change is the elevation of error-prone DNA polymerase, which has the ability to replicate through DNA damage^30^. As above, enzymes levels that are altered on the Day 30 vs. Day 60 list, but do not appear on the Day 15 vs. Day 30 list, may be due to a delayed effect of grape consumption.

### KEGG pathway analysis for all subjects

Finally, a comparison of KEGG (Kyoto Encyclopedia of Genes and Genomes) pathways (level 3) associated with the microbiome was investigated for Day 15 vs 30, Day 30 vs 60, and Day 15 vs 60. The results are shown in Tables 7, 8 and 9, respectively. Again, entries were selected on the basis of strong *p-*values as well as Cohen *d-*values indicating an effect size in the medium to large range. A relatively low number of pathway alterations were observed.

**Table 7:** KEGG pathways altered when comparing Day 15 vs. 30 for all subjects.

Pathway	Log2 (Fold-change)	*P* value	Cohen's *D*
Cysteine peptidases; family C56: PfpI endopeptidase family	0.176	0.057	0.511
ABC transporters, prokaryotic type; ABC-2 type and other transporters; hemin transporter [MD:M00257]	1.646	0.065	0.500
ABC transporters, prokaryotic type; ABC-2 type and other transporters; heme transporter [MD:M00259]	− 0.653	0.078	0.474
NarL family; UhpB-UhpA	− 0.636	0.091	0.455

**Table 8:** KEGG pathways altered when comparing Day 30 vs. 60 for all subjects.

Pathway	Log2 (Fold-change)	*P* value	Cohen's *D*
1. Oxidoreductases; 1.20 acting on phosphorus or arsenic in donors; 1.20.4 with disulfide as acceptor	0.503	0.015	0.666
ABC transporters, prokaryotic type; ABC-2 type and other transporters; teichoic acid transporter [MD:M00251]	0.393	0.037	0.567
Nonribosomal peptide synthetase (NRPS); linear NRPS; surfactin family lipopeptide synthetase	0.579	0.056	0.515
ABC transporters, prokaryotic type; monosaccharide transporters; d-allose transporter [MD:M00217]	0.487	0.062	0.501
Aspartic peptidases; family A31: HybD endopeptidase family	0.463	0.069	0.489

**Table 9:** KEGG pathways altered when comparing Day 15 vs. 60 days for all subjects.

Pathway	Log2 (Fold-change)	*P* value	Cohen's *D*
Anoxygenic photosystem; green sulfur bacteria; chlorosome	3.362	0.033	0.587
Nonribosomal peptide synthetase (NRPS); linear NRPS; Surfactin family lipopeptide synthetase	0.763	0.040	0.560
1. Oxidoreductases; 1.3 acting on the CH–CH group of donors; 1.3.7 with an iron-sulfur protein as acceptor	0.395	0.051	0.526
ABC transporters, prokaryotic type; ABC-2 type and other transporters; lipopolysaccharide transporter [MD:M00320]	− 0.172	0.053	0.519

Comparing Day 30 vs. 15 indicates enhancement of “Cysteine Peptidases” which play key role in hemoglobin hydrolysis, blood cell invasion, egress, and surface proteins processing^31^, both enhanced and reduced “ABC Transporters”, which participate in the movement of metabolites to the cell surface^32^, and reduction of “Narl Family”, which is a sensory transduction pathway^33^.

Comparing Day 60 vs. 30, reveals enhance “Oxidoreductases” (a large class of enzymes catalyzing the transfer of electrons^34^), “ABC Transporters”, “Nonribosomal peptide synthetase (NRPS)” (catalyze synthesis of important peptide products from a variety of standard and non-proteinogenic amino acid substrates^35^) and “Aspartic peptidases” (important metabolic processes in microorganisms^36^).

In the Day 60 vs. 15 comparison, “ABC pathways” are again reduced, and “NRPS” and “Oxidoreductases” enhancement is retained. The most prominent alteration found in this entire data set is “Anoxygenic photosystem” enhancement in this time period. This is somewhat surprising given that anoxygenic phototrophic bacteria are known for growth using energy from light without evolving oxygen. However, some organisms are known to grow aerobically in the dark and perform bioremediation of recalcitrant dyes, pesticides, and heavy metals under anaerobic conditions^37^.

### Plasma GC–MS metabolomics

#### Day 15 vs. Day 30 plasma analysis

**Figure 2:**
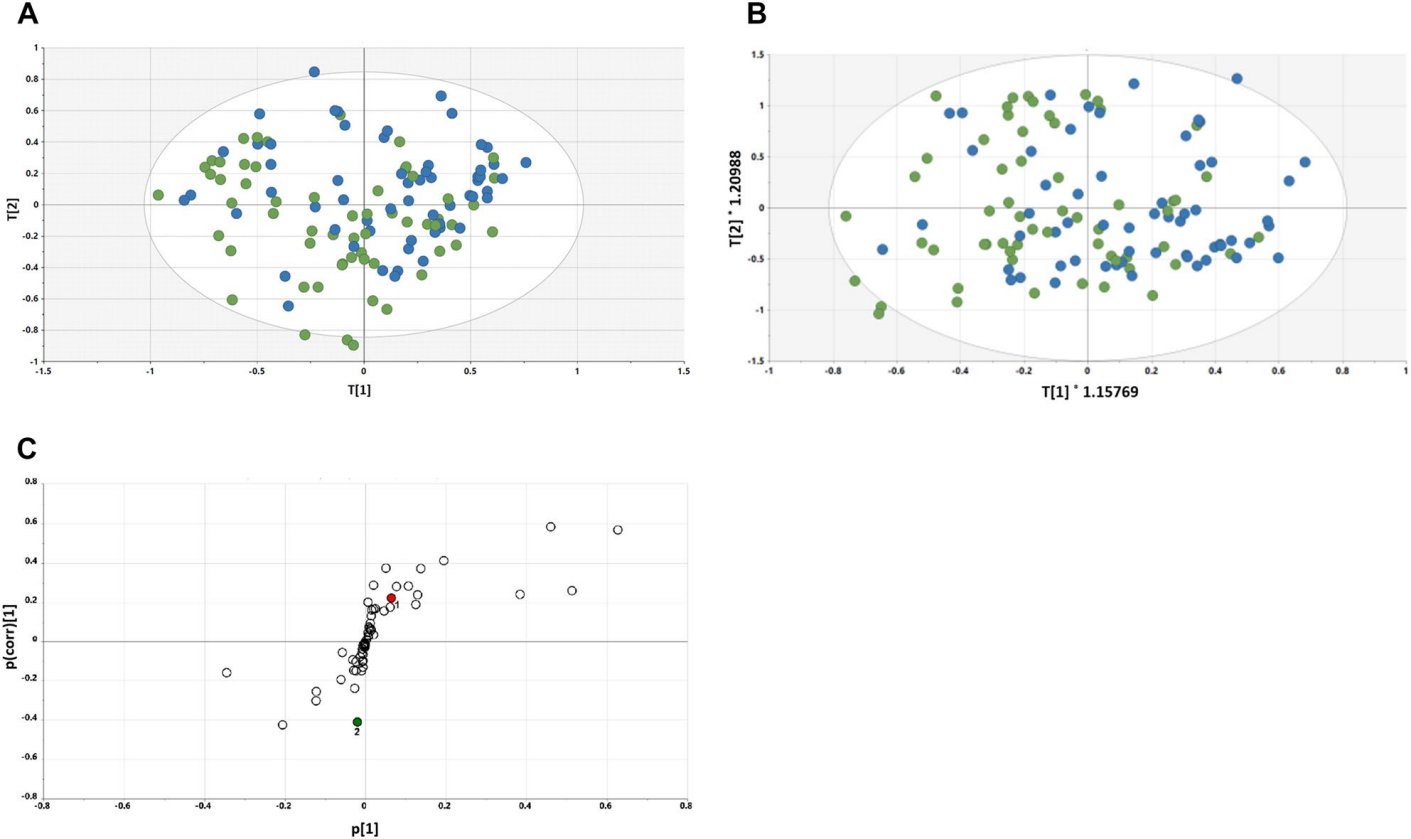
(**A**) PLS-DA scores plot for Day 15 (green) plasma vs. Day 30 (blue) plasma, (**B**) OPLS-DA scores plot for Day 15 (green) plasma vs. Day 30 (blue) plasma, and (**C**) OPLS-DA loadings S-plot for Day 15 plasma vs. Day 30 plasma. 1, Stearic acid; 2, Glucuronic acid.

The PLS-DA scores plot for Day 15 vs. Day 30 is shown in Fig. 2A. As can be seen, there is very little difference between the scores of the Day 15 (green) and Day 30 (blue) plasmas, with a tendency of Day 30 to move towards the upper right quadrant. The OPLS-DA scores plot for Day 15 vs. Day 30 is shown in Fig. 2B. Here, there is a slight tendency for the Day 15 samples to cluster to the left and the Day 30 samples to cluster to the right. However, there is clearly no separation between the two time points.

The OPLS-DA loadings S-plot displays the distribution of the 59 identified plasma metabolites (Fig. 2C). The elevated and depressed metabolites were all screened using univariate pairwise analysis for significant differences between the two study days. Only two plasma metabolites were found to be statistically significantly altered between the restricted diet (Day 15) and the grape diet (Day 30). Stearic acid was elevated 10% (P < 0.05) and β-d-glucuronic acid was depressed 17% (*p* = 0.003) in plasma.

#### Day 30 vs. Day 60 plasma analysis

**Figure 3:**
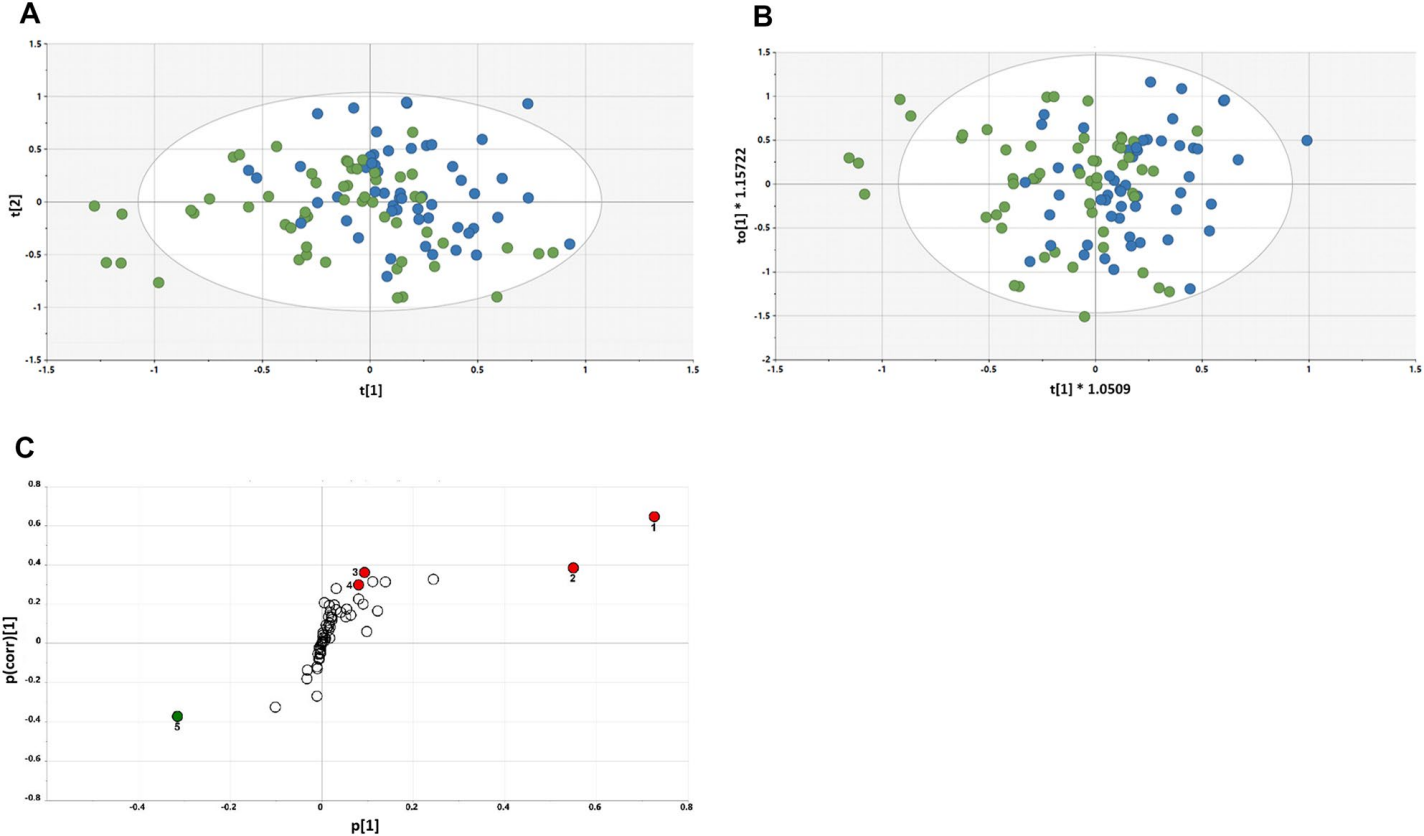
(**A**) PLS-DA scores plot for Day 60 (blue) plasma vs. Day 30 (green) plasma; (**B**) OPLS-DA scores plot for Day 60 (blue) plasma vs. Day 30 (green) plasma; (**C**) OPLS-DA loadings S-plot for Day 30 plasma vs. Day 60 plasma. 1, glucose; 2, mannose; 3, fructose; 4, 2-hydroxybutanoic acid; 5, lactic acid.

The PLS-DA scores plot for Day 30 vs. Day 60 is shown in Fig. 3A. Compared to the Day 15 and Day 30 analysis, there is a marginally better separation of scores between the Day 30 samples and the Day 60 samples, with a tendency of Day 30 to the left and Day 60 to the right. When an OPLS-DA model was generated (Fig. 3B), the separation appeared slightly better. This was reflected in the OPLS-DA loadings S-plot (Fig. 3C), where four metabolites were elevated in Day 60 plasma and one depressed. Interestingly, three sugars were elevated after changing from the grape diet back to the restricted diet—glucose (+ 11%, *p* = 0.007), mannose (+ 16%, *p* = 0.03) and fructose (+ 17%, *p* = 0.008), together with 2-hydroxybutanoic acid (+ 7%, *p* = 0.01). One metabolite was depressed in plasma after returning from the grape to the restricted diet—lactic acid (− 17%, *p* = 0.02).

### Urine GC–MS metabolomics

#### Day 15 vs. Day 30 urine analysis

**Figure 4:**
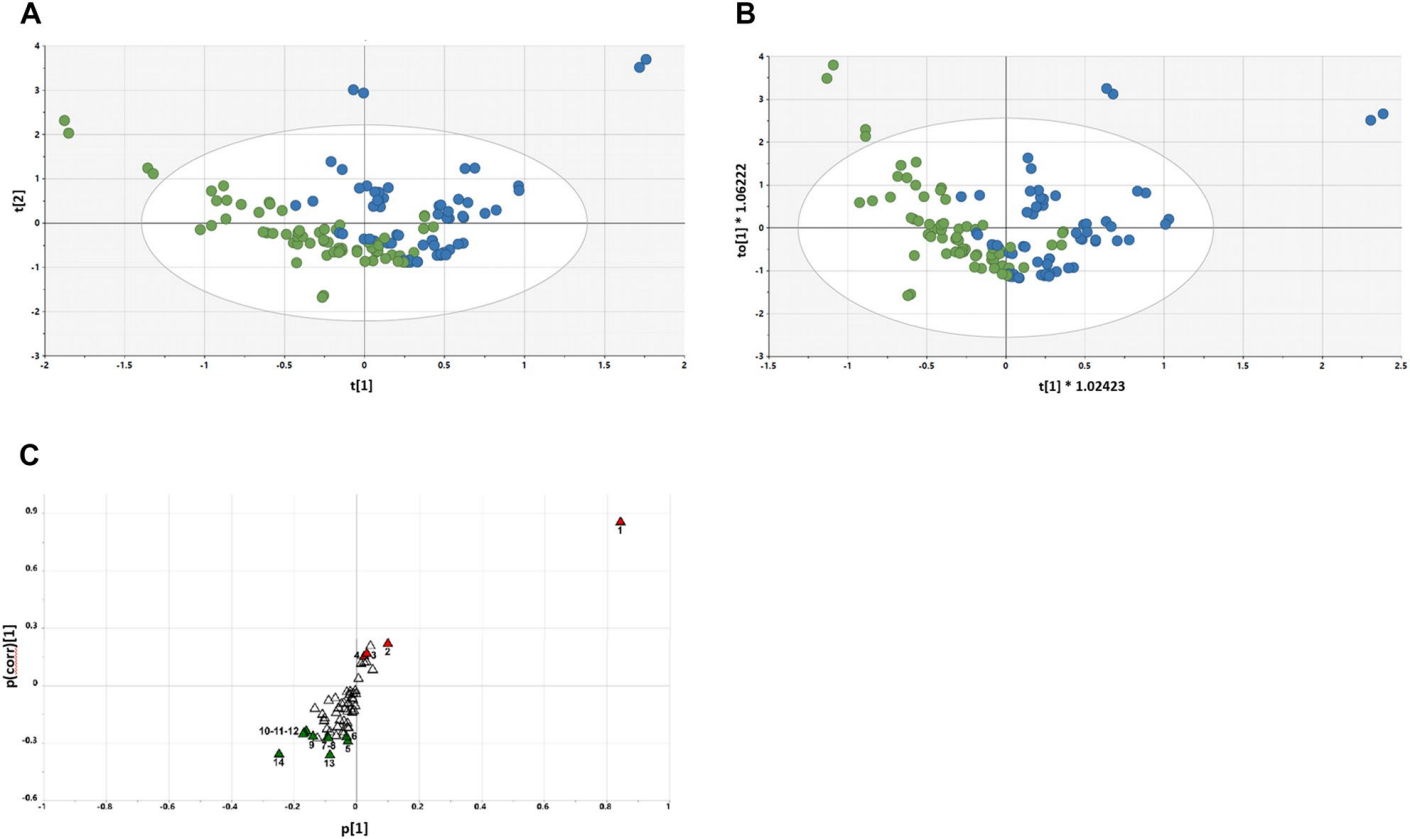
(**A**) PLS-DA scores plot for Day 15 (green) urine vs. Day 30 (blue) urine; (**B**) OPLS-DA scores plot for Day 15 (green) urine vs. Day 30 (blue) urine; (**C**) OPLS-DA loadings S-plot for Day 15 urine vs. Day 30 urine. 1, Tartaric acid; 2, 2′-Deoxyribonic acid; 3, Glutaconic acid; 4, 3-Hydroxyphenylacetic acid; 5, Valine; 6, 3-Indoleacetic acid; 7, Ribose; 8, 2,3-Dihydroxybutanoic acid; 9, Galactose; 10, Glucose; 11, Hippuric acid; 12, Carbamic acid; 13, Malonic acid; 14, Levoglucosan.

The PLS-DA scores plot for Day 15 urine vs Day 30 urine is shown in Fig. 4A. In this scores plot, a separation between the Day 15 and Day 30 urines emerges. When this was further examined using an OPLS-DA model (Fig. 4B), the same emerging difference between restricted and grape diet urines was observed. The OPLS-DA loadings S-plot (Fig. 4C) revealed four elevated urinary metabolites and ten diminished metabolites. These differences were more profound than those seen in the comparative plasma samples.

#### Day 30 vs. Day 60 urine analysis

**Figure 5:**
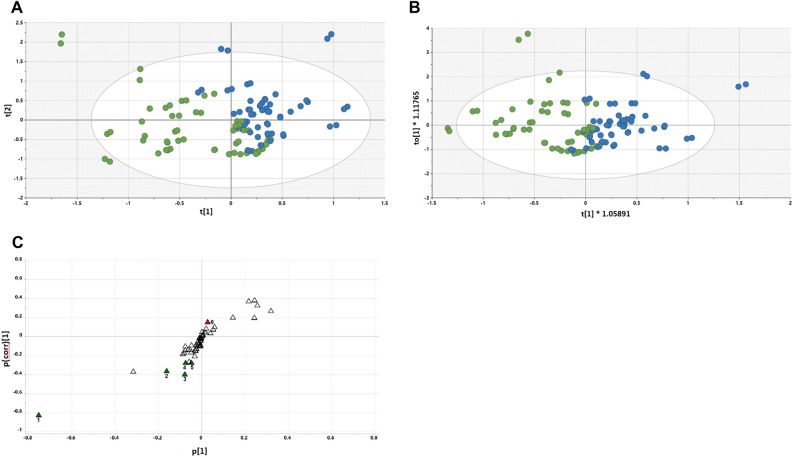
PLS-DA scores plot for Day 30 (green) urine vs. Day 60 (blue) urine (**A**); OPLS-DA scores plot for Day 30 (green) urine vs. Day 60 (blue) urine (**B**); OPLS-DA loadings S-plot for Day 30 urine vs. Day 60 urine. 1, Tartaric acid; 2, 2′-Deoxyribonic acid; 3, Glutaconic acid; 4, Ribonic acid; 5, 3-Hydroxyphenylacetic acid; 6, Fumaric acid (**C**).

The PLS-DA scores plot for Day 30 urine vs Day 60 urine is shown in Fig. 5A. The restricted and grape diet urines are almost completely separated in the PLS-DA scores plot, and continued improvement was realized when the OPLS-DA model was applied (Fig. 5B). As shown in the OPLS-DA loadings S-plot (Fig. 5C), one urinary metabolite was elevated by return to the restricted diet and five metabolites were diminished.

### Taxonomic and metabolomic analysis for selected subjects

**Figure 6:**
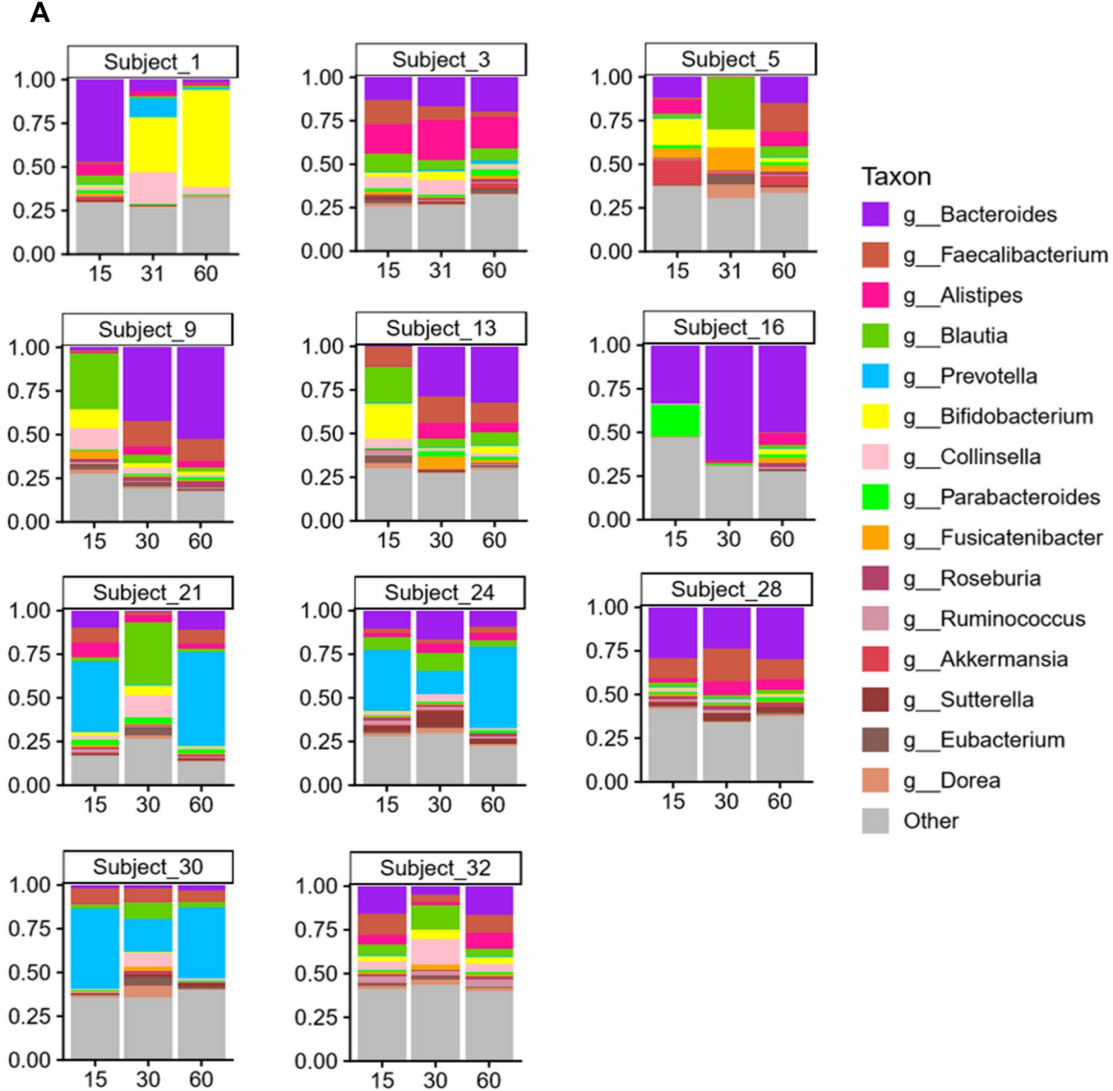

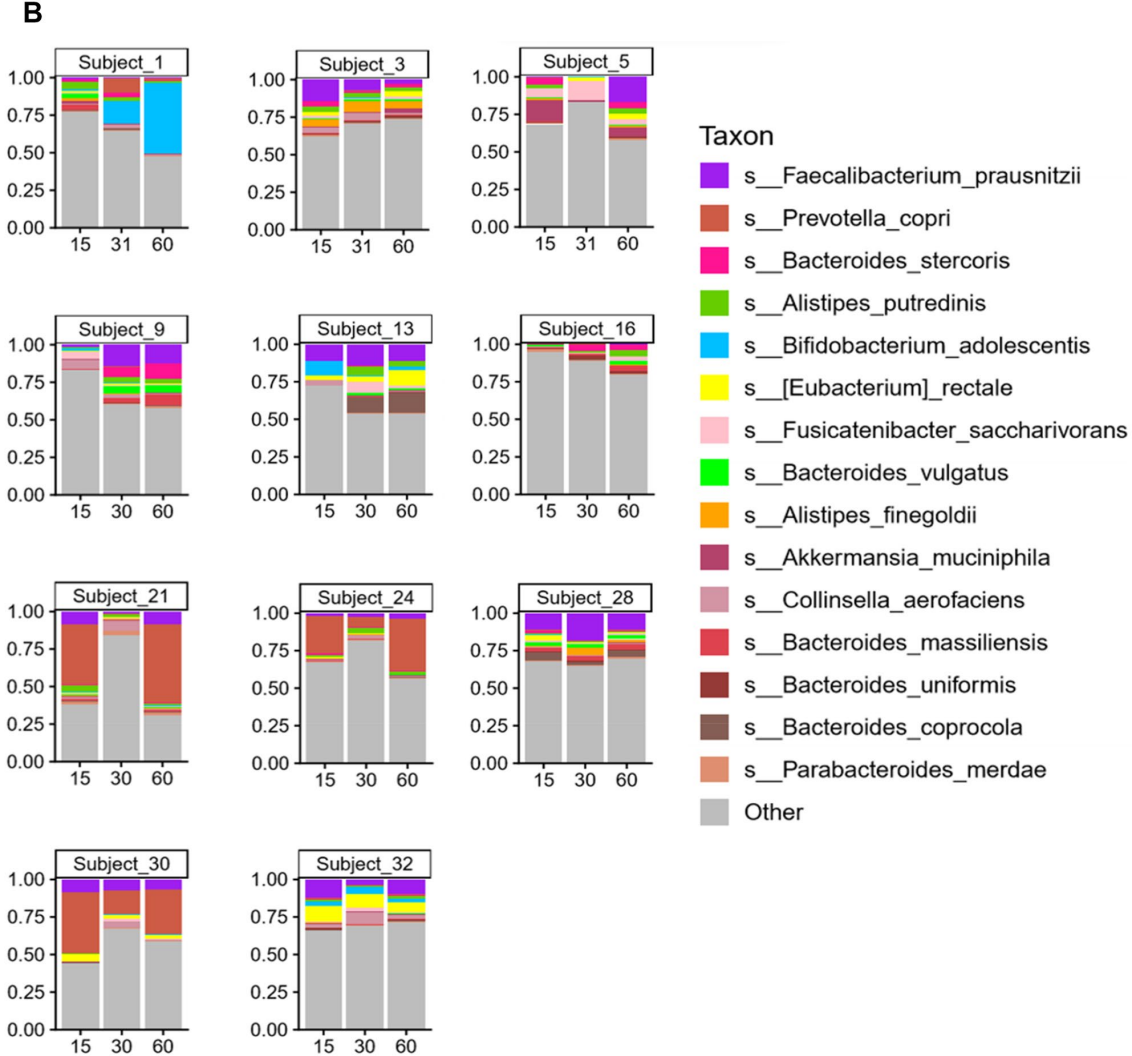
Arbitrary selection of 11 subjects based on unique profile shifts of genus (**A**) and species (**B**).

Lastly, we thought it would be of interest to segregate a group of subjects showing the most obvious unique profile shifts based on bacterial genus and species, and compare these subjects with the remainder of the study population. Accordingly, 11 subjects were selected demonstrating the profile shifts shown in Fig. 6.

As expected, based on the method of selecting these 11 subjects, comparison of alpha-diversity (Supplement 1, Fig. S1D) and beta-diversity (Supplement 2, Fig. S1D) with the remaining 18 subjects, revealed no significant differences. However, as summarized in Supplement 3, comparison of Day 15 vs Day 30, and Day 15 vs Day 60, of these 11 subjects with the remaining 18 subjects, revealed significant differences in terms of taxonomic, enzyme and KEGG pathway comparisons. For example, in the 15 vs. 60 day comparison, the abundance of Erysipelotrichia, Erysipelotrichales, *Erysipelotrichaceae*, and *Ruminococcus*, were altered (Supplement 3, Table S1), as were three KEGG pathways (Supplement 3, Table S2) and 13 enzymes (Supplement 3, Table S3).

Comparison of Day 30 vs Day 60 (Supplement 3, Table S4) revealed the following taxa to be significantly different—class: Coriobacteriia (bile acid metabolism^38^), order: Coriobacteriales (bile acid metabolism^39^), family: *Coriobacteriaceae* (bile acid metabolism^40^), genus: *Collinsella* (Collinsella has been linked to pro-inflammatory dysbiosis in type 2 diabetes and with circulating insulin suggestive of a mechanism for promotion of NAFLD pathology^41^), species: *Collinsella aerofaciens* (*C. aerofaciens* is the major utilizer of lactose in the human colon. Several studies demonstrated that *Collinsella* and *Bifidobacterium* can modify the host bile acids to modulate the virulence and pathogenicity of enteric pathogens^42^). Correspondingly, several significant differences were observed in the Day 30 vs. 60 comparisons of enzymes and KEGG pathways (Supplement 3, Tables S6 and S7).

The rift in taxonomic abundance variation was less profound in the Day 15 vs. 60 comparison of the select subjects with the remainder of the group (Supplement 3, Table S7). However, many significant differences were observed in enzyme levels (Supplement 3, Table S8) and KEGG pathways (Supplement 3, Table S9).

**Figure 7:**
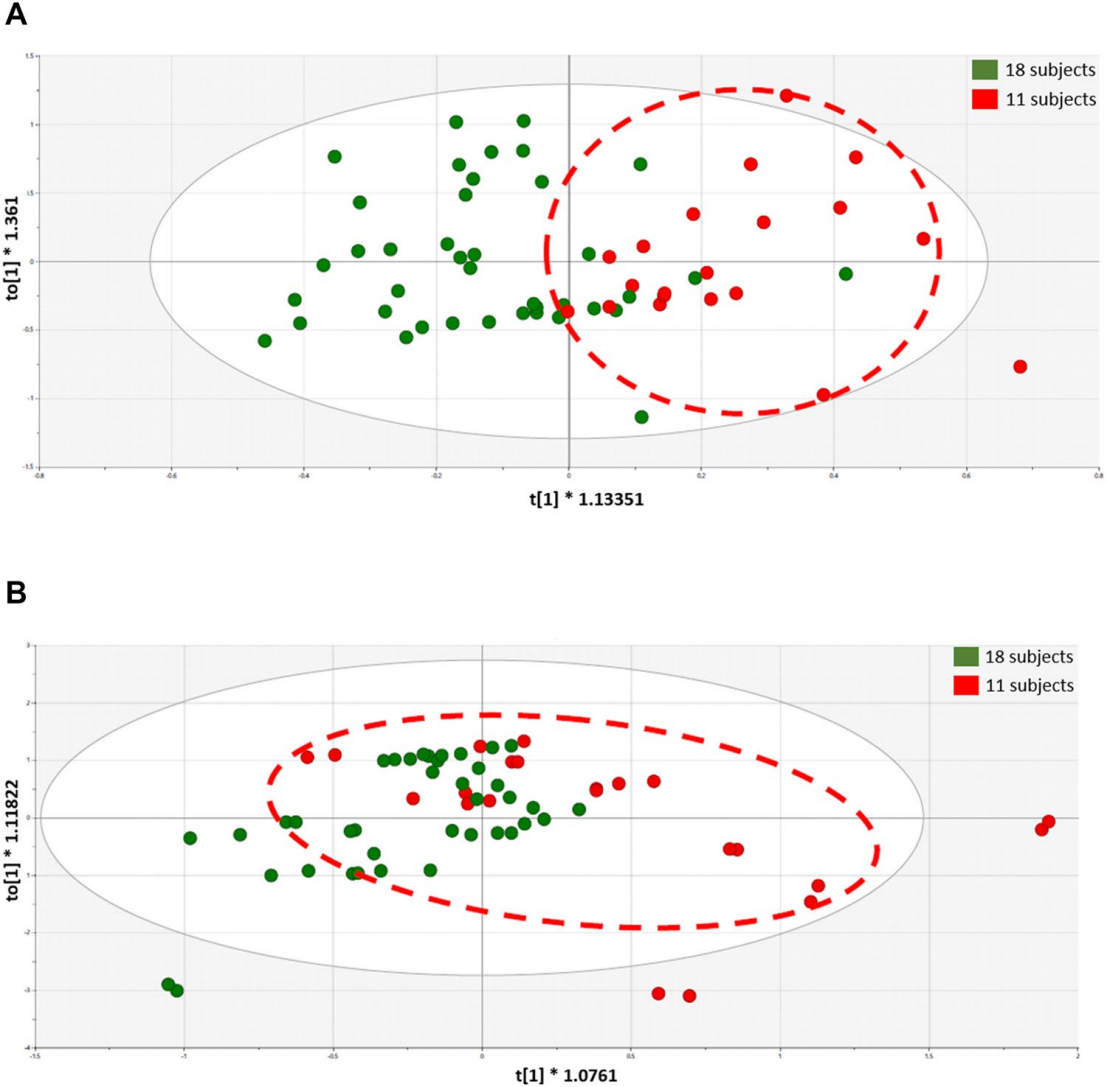
Plasma sample OPLS-DA scores plots of the selected group of 11 subjects (red) versus the remaining 18 subjects (green) determined with plasma (**A**) and urine (**B**) samples.

Finally, comparative metabolomic analyses of the two subgroups were investigated with OPLS-DA at the Day 30 (following grape consumption) time point. As shown in Fig. 7, the selected group of 11 subjects can be distinguished from the remaining 18 subjects on the basis of OPLS-DA scores plots, with both urine and plasma samples. However, univariate data analyses of plasma and urinary metabolites judged to originate from the microbiota did not show great difference between the two groups, aside from *myo*-inositol in plasma, which was somewhat decreased (*p* = 0.03) (Supplement 4).

## Discussion

Whereas the intestinal microbiome of a healthy individual is relatively stable, dysbiosis may lead to or be associated with health problems such as Crohn's disease, autoimmune diseases, colon cancer, gastric ulcers, cardiovascular disease, and obesity. In the present study, the human subjects varied by gender and age, but all were deemed to be in good health. It was thereby expected these individuals began the study in a eubiotic state, i.e., with a balance between beneficial and harmful bacteria. Thus, our goal was not geared toward inducing any particular alteration. Our goal was simply to determine if consumption of a common dietary fruit, i.e., the grape, functioned as a prebiotic, probiotic, or antibiotic, or, in fact, led to no major change at all. In so doing, following a two-week period on a controlled dietary regimen, the diet was supplemented for a two-week period in which a well-defined grape surrogate equivalent to three normal servings were consumed on a daily basis, and finally, a one-month washout period in which the controlled dietary regimen was continued but grape supplementation was discontinued. Fecal samples were collected for analysis at each of these three time points.

We first examined alpha-diversity as a broad indication of richness (number) or evenness (relative abundance) of the entire study population. There was no perceptible differences between the populations on Day 15, 30 or 60 (Supplement 1, Fig. S1A). Given that alpha-diversity may vary with gender and age^43^, we segregated our subjects into cohorts and repeated the analysis. Again, no major differences were observed at the various time points, other than with females, from the age of 29 to 39 years, in comparing the baseline to day 30 (15 days of consuming grapes) where Cohen's *D* value for the Chao test was 0.836 and the *p* value was 0.114 (Student’s paired *t*-test), and comparing the baseline (Day 15) with the washout period (Day 60), where Cohen's *D* value was 0.982 with a corresponding *p* value of 0.079 (Supplement 1, Fig. S1B). This difference was not observed with male subjects (Supplement 1, Fig. S1C), nor was there a difference when comparing day 30 and day 60 of the female group (Chao test; Cohen’s *D* value 0.047 and *p* = 0.93, Student’s paired *t*-test).

It is interesting that the alpha-diversity of the females showing a change following grape consumption did not return to baseline after the 30-day washout period. The ramifications of this shift, if any, are worthy of further investigation. We note that Yang et al.^15^ reported a significant change in the Shannon index when 19 human subjects were provided with the grape surrogate used herein for a period of 4 weeks. The gender of the subjects enrolled in this study was not specified, although it was stated that the age range was 18–55 years and postmenopausal women were excluded. We also note that higher dietary quality may be reflected in a higher gut microbiota diversity^44^, as was observed with the female group of our study.

Next, we investigated beta-diversity, similarity or dissimilarity, at Day 15, 30 and 60. Bray–Curtis dissimilarity was calculated and visualized via PCA and PCoA. As illustrated in Supplement 2, there was no major difference observed with the study population prior to, during, or after grape consumption. This was true for the study population as-a-whole, as well as subdivisions based on gender.

Thus, based on both alpha- and beta-diversity analyzes, we conclude grape consumption per se does not alter the overall communal relationships of the microbiota with this study population. Unlike controlled animal or in vitro studies, significant inter-individual variation exists when examining free-living human beings. This is illustrated by comparison of the first of the triplet bars shown in Fig. 1A (Day 15) and by the composite overlays illustrated in Fig. 1B. Following the period of grape intervention (Day 30), comparison of the first two bars of Fig. 1A (Day 15 vs. Day 30) suggests changes in essentially every case, as can be further visualized by comparison of the composite overlays of the corresponding days (Fig. 1B). Based on the study design, the most likely factor leading to these changes was the 2-week administration of dietary grapes. The final bars of the triplets illustrate the status of the microbiota after termination of grape administration, as does the third overlay. The question addressed by this final time point was if the pattern would return to that preceding grape administration, or would any changes be perpetuated. The answer seems to reside with the individual.

First of all, it is clear that the abundance of components of the microbiota does in fact change with dietary grape intervention, as can be perceived from the composite overlays at the various time points (Supplement 3, Fig. S1). Differences exist between all three time points. A more granular examination with select subjects is illustrated in Fig. 6. What we can glean from these examples is that a shift in the microbiome induced by grape consumption may be durable, as shown by subjects 1, 9, and 13, may return to the baseline of 15 days, as shown by subjects 21 and 32, show no overt change (subjects 3 and 28), or something in between.

Comparative analyses of the taxonomy of the entire study population indicated significant changes in microbial abundance when comparing Day 15 vs. Day 30 (Table 1), Day 30 vs. Day 60 (Table 2), and Day 15 vs. Day 30 (Table 3). Corresponding changes in enzyme levels (Tables 4, 5, 6) and KEGG pathways (Tables 7, 8, 9) were associated with the taxonomic shifts at each time comparison. As an attempt to decipher the physiological consequences of these effects, metabolomic analyses were performed with plasma and urine specimens provided by the subject population at each time point for the investigation of metabolites deemed to be associated with the microbiome.

With plasma, PLS-DA scores plot for Day 15 vs. Day 30 showed little difference (Fig. 2A). Similarly, the OPLS-DA scores plot revealed no clear separation between the two time points (Fig. 2B). Consistent with this, the OPLS-DA loadings S-plot displayed the distribution of the 59 identified plasma metabolites (Fig. 2C), but only two [stearic acid elevated 10% (*P* < 0.05) and β-d-glucuronic acid depressed 17% (*p* = 0.003)] showed statistically significant changes.

When comparing Day 30 vs. Day 60, relative to the Day 15 vs. Day 30 comparison, marginally better separation was observed in the PLS-DA scores plot (Fig. 3A), which was somewhat improved by generation of an OPLS-DA model (Fig. 3B). As reflected in the OPLS-DA loadings S-plot (Fig. 3C), three sugars were elevated after changing from the grape diet back to the restricted diet [glucose (+ 11%, *p* = 0.007), mannose (+ 16%, *p* = 0.03) and fructose (+ 17%, *p* = 0.008)], together with 2-hydroxybutanoic acid (+ 7%, *p* = 0.01). Lactic acid was depressed in plasma after returning from the grape to the restricted diet (− 17%, *p* = 0.02).

The increase in glucose, mannose and fructose after grape cessation during the washout period after grape cessation correlates with the potential of grape consumption to be of benefit in metabolic syndrome, as reported in the literature^45,46^. Consistent with this suggestion, we have reported the addition grapes to both a standard diet and a high-fat diet in mice is associated with an up-regulation of the malate-aspartate shuttle and therefore the efficiency of glucose utilization by the liver^12^. Moreover, 2-hydroxybutanoic acid is primarily produced in the liver during glutathione synthesis, which we have also reported to be elevated in relation to a grape diet in mice^6^.

Analysis of urine collected over a 24 h period revealed more striking differences when comparing the time points of the entire subject population. Clear separation of Day 15 urine vs Day 30 urine is illustrated by the PLS-DA scores plot (Fig. 4A) and OPLS-DA model (Fig. 4B). The OPLS-DA loadings S-plot (Fig. 4C) revealed four elevated urinary metabolites, the enhancement of which was considered a direct result of grape consumption, in that they were all reduced after an additional 30 days on a grape-free diet.

Tartaric acid was most profoundly increased (fivefold, *p* < 0.0001). As a component of the grape itself, this can be viewed as a good indicator for monitoring grape consumption or dietary compliance. Also elevated by consumption of the grape diet was 2’-deoxyribonic acid (+ 26%, *P* = 0.009). This metabolite is derived by the hydrolysis of 2′-deoxyribonolactone, which is formed in DNA by the oxidation of 2′-deoxyribose at abasic sites, the most frequent DNA lesion occurring at a rate of thousands per cell per day. This process occurs preferentially at sites of DNA replication where the lagging DNA single strand is most vulnerable, particularly to depurination^47^. 2′-Deoxyribonolactone is released from duplex DNA with a half-life of 32 to 54 h, but faster from single-stranded DNA with a half-life of approximately 20 h^48^. Therefore, the elevation of 2′-deoxyribonic acid after grape consumption may be an accumulation of events occurring over many preceding days. It is also possible that this process represents naturally occurring DNA depurination whose repair is enhanced by one or more grape constituents. Furthermore, in two previously reported investigations of grape administration to mice, we also observed elevations of 2′-deoxyribonic acid that were associated with administration of a grape diet^6,12^, and inter-individual variation was observed in human intervention studies^49^.

Further, glutaconic acid, another 5-carbon compound derived from glutamate, was also found to be elevated after grape consumption (+ 26%, *p* = 0.004). 3-Hydroxyphenylacetic acid, related to tyrosine metabolism, was also elevated (+ 44%, *p* = 0.002). On the other hand, urinary excretion of two substances related to gut microbiota metabolism, 3-indoleacetic acid and hippuric acid, was diminished, as well as valine, ribose, 2,3-dihydroxybutanoic acid, galactose, glucose, carbamic acid, malonic acid, and levoglucosan.

A survey of the literature revealed the bacterial origin of glutaconic acid involves *Clostridium symbiosum*^50^ and the bacterial origin of 3-hydroxyphenylacetic acid involves *Parabacteroides* spp.^51^, *Clostridia* spp.^52^, or *Klebsiella pneumoniae*^53^. These species are not included in Table 1 which summarizes major taxonomic alterations and, in fact, subsequent analyses revealed these species were not significantly different on Day 15 vs. Day 30 in this study population (Supplement 5). Further work is required to clarify such issues, but this dichotomy does illustrate the value of metabolomics over microbiomics in defining the actual production of potentially bioactive metabolites.

Analysis of PLS-DA scores plot for Day 30 urine vs Day 60 urine (Fig. 5A) and OPLS-DA modeling (Fig. 5B) showed even more distinct separation of scores. In the OPLS-DA loadings S-plot (Fig. 5C), one urinary metabolite was elevated by return to the restricted diet and, as noted above, metabolites following the addition of grape to the diet were diminished.

Finally, as mentioned above and illustrated in Fig. 6, it is clear that individual variation will be discerned in a free-living group of human beings. For the purpose of this study, the subject population agreed to dietary and lifestyle restrictions (Supplements 6 and 7) in order for us to access the impact of grape consumption as accurately as possible. This is not to say, however, that we expected individuals to respond in a completely homogeneous manner. Based on these considerations, we thought it would be of interest to segregate a specific segment of the study population, initially based on taxonomic profiles of the microbiome, and compare this select group with the others participating in the study using the same methodology.

Of the 29 participants who completed the study, 11 subjects were selected based on unique profile shifts of genus and species when comparing the three time points (Fig. 6). As with the group as a whole, no significant differences were found in alpha- and beta-diversity of the microbiome of the 11 select subjects. However, at each time comparison (15 vs. 30; 15 vs. 60; 30 vs. 60), significant differences were uncovered when the select 11 subjects were compared with the remaining 18 subjects in terms of taxonomy, enzyme levels and KEGG pathway analysis (Supplement 3). Confirming the functional significance of these differences, at the Day 30 time point (following grape consumption), clear separation of was observed using OPLS-DA, both with plasma samples (Fig. 7A) and urine (Fig. 7B). The complete chemical nature of these metabolic changes remains to be characterized. Thus far, our analysis of plasma and urinary metabolites judged to originate from the microbiota has only yielded the identity of *myo*-inositol in plasma, which was somewhat decreased (*p* = 0.03) (Supplement 4).

## Conclusions

In sum, the data presented herein demonstrate that grape consumption does not perturb the eubiotic state of the microbiome with normal, healthy human subjects. Grape consumption does change taxonomic composition of the microbiome, enzyme levels, KEGG pathways, and the metabolome. As is common with work involving a heterogeneous group of free-living human beings, inter-individual variation was demonstrated by sub-analysis of the study population. However, with the study group as a whole, changes mediated by grape consumption were observed which may be expected to broadly apply. Further research is required to determine if these responses are responsible for or related to any of the health benefits that have been associated with grapes, although it does seem logical to expect changes of these types are profound enough to have significance.

## Methods

### Study procedure: plasma, urine, and fecal sample collection

The objective of this study was to determine the potential effect of grape consumption on the microbiome, based on the microbial composition of the microbiome and metabolomics analyses. As an attempt to remove as many confounders as practicable, subjects were required to meet the Inclusion/Exclusion Criteria described in Supplement 6. With subjects meeting the criteria, the overall nature of the study was discussed. In particular, the diet and supplement restrictions associated with the study (described in Supplement 7) were reviewed, and subjects were given a contact number to ask any questions related to allowable food. At this point, subjects signing an Informed Consent document were enrolled in the study.

#### Day 1

On Day 1 of the study, subjects were provided with the first fecal sample collection kit, and started the study diet. They were instructed to collect a fecal sample on Day 13/14 and return the test kit containing the sample on Day 15 (± 2 days). In addition, the subjects were instructed to fast for a 12 h period prior to returning the fecal sample on Day 15, in that plasma would be prepared on that day as well.

On Day 1 of the study, subjects were also provided with a urine collection vessel. On or about day 14 of the study, each volunteer collected a complete 0–24-h urine sample. During the collection period at home, the sample was refrigerated between and after each collection.

#### Day 15

On day 15 of the study, the first fecal test kits were received. All fecal samples were collected, recorded, and prepared for shipment according to Diversigen guidelines. Within 24 h, samples were sent to Diversigen (New Brighton, MN) for analysis by express mail.

In addition, the first urine sample bottles were received and shaken by hand to ensure homogeneity. Volume was measured in mL and recorded. Five mL were transferred to15-mL Falcon tubes, labelled with the subject’s code, date and time, and 24-h urine volume, and frozen at − 20 °C prior to analysis.

Subjects were provided with the second fecal test kit, a second urine collection vessel, and sufficient grape powder to last the duration of the two-week grape powder consumption time period. In addition to providing instruction sheets (Supplement 8), the subjects were instructed on how to use the powder during the study. Just prior to consumption, the subjects mixed the grape powder (36 g) with approximately 6 oz of water. This was done two-times per day (once in the morning and in the afternoon/evening) for 14 days. Subjects were advised if a dose were missed that they should take it as soon as possible. Subjects were given a daily diary in which to record product usage and were instructed to bring in the empty powder pouches to be recorded for compliance on Day 30 (± 2 days).

Finally, at least 7 mL of whole blood were drawn into a 10 mL tube heparinized Vacutainer (contained sodium heparin) and placed on ice until centrifugation at 4 °C for at least 15 min at 2200–2500 rpm. Using a glass Pasteur pipette (one time use only), the upper plasma layer (3 mL) was carefully removed without transferring any red cells and placed in a plastic screw-top tube, labeled with subject’s code, date and time. Samples were storied at − 20 °C prior to analyses.

#### Day 30

On day 30 of the study, the subjects returned the second fecal test kit containing a specimen that had been collected in the recent past, and a 24-h urine collection. Samples were processed and stored as described above. Empty pouches that had previously contained the grape powder were returned and the subjects were interrogated in regard to their experience. The adverse events were reported.

Whole blood was collected and plasma was prepared as described above. Prior to each blood collection, subjects confirmed that had fasted for at least the past 12 h. Subjects were given the third fecal sample collection kit and the third urine collection vessel, and they continued the grape-free study diet and the drug-free regimen for a four-week washout period.

#### Day 60

On Day 60 (± 2 days), subjects returned their final fecal and urine samples which were prepared for analysis, and the final plasma samples were collected.

### Grape powder

To assure the consistency and continuity of experimental and clinical research concerning the biological and physiologic potential of grapes, a freeze-dried powder is manufactured under the auspices of the California Table Grape Commission (Fresno, CA)^54^. The grape powder, which serves as a surrogate for fresh grapes, is composed of fresh seeded and seedless red, green and black grapes that are ground and freeze-dried to retain their bioactive compounds. The powder is prepared using good manufacturing procedures for foods. Further quality assurance is provided by assuring the product is contaminant-free through microbial analyses. In addition, the product is subjected to chemical standardization for the quantitation of key phytochemical constituents^54^. For the current studies, vacuum-sealed packets containing 36 g of standardized freeze-dried grape powder were supplied and stored at − 20 °C until use.

### Human subjects

#### Human subject demographics

Forty-one (41) subjects were enrolled in the study and 29 subjects (70.7%) completed the study. Twelve (12) subjects (29.3%) discontinued the study. Five (5) subjects (12.2%) withdrew their consent, 3 subjects (7.3%) were lost to follow-up, 2 subjects (4.9%) were due to AEs (exclusionary medication needed and Covid-19 symptoms) and 2 subjects (4.9%) were due to Other (exclusionary medication and positive for Covid-19).

The mean (standard deviation [SD]) subject age was 39.8 (9.6) years, where the median age was 40.0 years, and the minimum and maximum ages were 20.9 to 55.7, respectively. Twenty-two (22) subjects (53.7%) were female, and 19 subjects (46.3) were male. Subject race included White/Caucasian (40 subjects, 97.6%) and Other (1 subject, 2.4%). The majority of subjects, 29 (70.7%), were not Hispanic or Latino and 12 (29.3%) were Hispanic or Latino.

#### Institutional review board

The study protocol, the subject information and informed consent form (ICF), and other written subject information, were reviewed and approved by the Institutional Review Board (IRB) IntegReview, 3815 S. Capital of Texas Hwy, Suite 320, Austin, TX 78,704, Phone: 512–326-3001, Fax: 512–697-0085, http://www.integreview.com.

All research was performed in accordance with relevant guidelines and regulations; informed consent was obtained from all participants.

### Treatment of fecal microbiota and microbiome analysis

#### DNA extraction

Samples were extracted with PowerSoil Pro (Qiagen) automated for high throughput on the QiaCube HT (Qiagen), using Powerbead Pro Plates (Qiagen) with 0.5 mm and 0.1 mm ceramic beads.

#### DNA quantification QC

Samples were quantified with Quant-iT PicoGreen dsDNA Assay (Invitrogen).

#### Library preparation & sequencing

Libraries were prepared with a procedure adapted from the Illumina DNA Prep kit (Illumina). For BoosterShot® (Shallow Sequencing, 2 M reads/sample), libraries were sequenced on an Illumina NovaSeq using single-end 1 × 100 reads (Illumina).

#### Sequence quality control

DNA sequences were filtered for low quality (Q-Score < 30) and length (< 50), and adapter sequences were trimmed using cutadapt. Human host sequence reads were removed using Bowtie2.

#### Taxonomic annotation

Sequences were trimmed to a maximum length of 100 bp prior to alignment and converted to a single fasta using shi7. DNA sequences were aligned to a curated database containing representative genomes in RefSeq for bacteria with additional manually curated strains (Venti). Alignments were made at 97% identity against all reference genomes. Every input sequence was compared to every reference sequence in Diversigen’s Venti database using fully gapped alignment with BURST. Ties were broken by minimizing the overall number of unique Operational Taxonomic Units (OTUs). For taxonomy assignment, each input sequence was assigned the lowest common ancestor that was consistent across at least 80% of all reference sequences tied for best hit. Samples with fewer than 10,000 sequences were also discarded. OTUs accounting for less than one millionth of all species-level markers and those with less than 0.01% of their unique genome regions covered (and < 1% of the whole genome) were discarded. The number of counts for each OTU was normalized to the average genome length. Count data were then converted to relative abundance for each sample. The normalized and filtered tables were used for all downstream analyses.

#### Functional annotation

Kyoto Encyclopedia of Genes and Genomes Orthology groups (KEGG KOs) were observed directly using alignment at 97% identity against a gene database derived from the Venti strain database. To construct this database, a representative strain for each species in the Venti database was annotated using Prokka (v 1.12). Prokka annotations were cross referenced to KEGG IDs, and gene sequences with KEGG annotations were retained for use in the functional database. The KO table and downstream tables contain the directly observed KO counts converted to relative abundance within a sample. KOs were collapsed to level-2 and -3 KEGG pathways and KEGG Modules (www.kegg.jp/kegg/kegg1.html).

#### Alpha- and beta-diversity

The Chao1 index, Shannon Index and observed OTU count (taxonomic group) were calculated using a rarefied, filtered taxonomy table set to the minimum depth allowed for a sample (10,000) using QIIME 1.9.1. Bray–Curtis beta diversity metrics were calculated from the filtered taxonomy and KEGG module/enzyme relative abundance using QIIME 1.9.1.2.

### Urine and plasma metabolomics

#### Plasma GC–MS metabolomics

First, 24 quality control (QC) samples were prepared by pooling 50 μL aliquots of all the plasma samples. Because multiple assays were to be conducted on each sample and to avoid repetitive freezing and thawing, every sample was divided into 200 μL aliquots and stored in Eppendorf tubes. Samples were labelled 1A, 1B, 1C, 2A, 2B, 2C…, where # = subject ID and A = Day 15 (± 2 days), B = Day 30 (± 2 days) and C = Day 60 (± 2 days). Samples were stored at − 20 °C. All samples were analyzed in duplicate and together with the QC samples. These were analyzed on seven consecutive days in lots of 30 and in an order that had been randomized. Most commonly, therefore, duplicates of plasma samples were analyzed on different days. Samples were derivatized with BSTFA/TMCS and analyzed with an Agilent GC–MS system using our previously described methods^12^. Chromatographic peaks were identified using AutoQuant (Agilent) that compared their mass spectra with the NIST 14 spectral library of 242,466 mass spectra. In cases of ambiguity where related metabolites produced similar mass spectra, for example the isomeric sugars glucose, galactose, fructose and mannose, authentic standards were employed and the peaks identified from their retention times on the gas chromatographic column. Some components produced more than one derivative (and therefore chromatographic peak), which were summed. The relative concentration of each metabolite was determined from the ratio of its peak area to the peak area of the internal standard 4-chlorophenylacetic acid. An Excel spreadsheet was then constructed using Quant Browser that contained the peak area ratio (relative concentration) of all identified metabolites in all samples. This data matrix was imported into SIMCA 17 in order to conduct multivariate data analysis.

#### Urine GC–MS metabolomics

First, because of its well established predominance in GC–MS chromatograms of derivatized urine, urea was removed from all urine samples by incubation with Jack bean urease. This procedure had been reported to increase the number of metabolites detected in urine and reduce their coefficient of variation^55^. In all other respects, the urine samples were analyzed over seven consecutive days by the same procedures as the plasma samples.

#### Partial least squares-discriminant analysis (PLS-DA)

This analysis is also known as projection to latent structures-discriminant analysis. It is a supervised multivariate data analysis, meaning that the class of each sample is included in the analysis. PLS-DA and other supervised methods are readily subject to over modeling of the data. To intercept such over modeling, PLS-DA models were subjected to a validation methodology using a leave-one out protocol with 200 permutations. Decay of the R2 (correlation) and Q2 (predictability) values to below 0.3 and 0, respectively, gives assurance that the data were not over modeled in the PLS-DA analysis.

#### Orthogonal PLS-DA analysis (OPLS-DA)

This analysis reduces the dimensionality of the PLS-DA scores plots. Consequent OPLS-DA loadings S-plots show the metabolites determined by GC–MS in relation to their relative abundance (X-axis) and their correlation to the OPLS-DA model (Y-axis). Loadings in the upper right quadrant represent metabolites that are upregulated in the test group and those in the lower left quadrant represent metabolites that are downregulated in the test group. Loadings that straddle the graphical point (0,0) represent metabolites that are unrelated to the experimental manipulation, e.g., change of diet.

### Arbitrary segregation specimen analyses

After generating the data sets described above, visual inspection of the stacked plots of microbial species showed some unique individual profile shifts (Figs. 1A and 6A). This, in combination with the highest differences observed in the geometric coordinates of PCA plots [Supplement 2, Fig. S2 panels D(i)-D(vi)] led to the selection of 11 subjects (six females and five males). Sub-analyses were performed comparing these 11 select subjects with the 18 remaining subjects using the same methodology as described above.

### Statistical analyses

To determine the statistical significance between the groups over time, paired *t*-tests were performed using Microsoft Excel. In addition, Cohen *d* values (effect size) were computed to compare different groups^56^. Univariate data analysis (Wilcoxon matched-pairs signed rank test) was performed using GraphPad Prism 9.3.1 for analyzing GC–MS metabolomics. Statistical significance was defined at a level of *p* < 0.05, although in some cases, *p* values in the range of 0.05–0.10 are reported along with Cohen’s *D* effect size^56^. Additional methods of statistical analyses are included in the text.

### Ethics approval and consent to participate

With subjects meeting the enrollment criteria, the overall nature of this study was discussed in detail. This included diet and supplement restrictions associated with the study. Subjects were given a contact number to ask any questions. Only subjects signing an Informed Consent document were enrolled in the study.

The study protocol, the subject information and informed consent form (ICF), and other written subject information, were reviewed and approved by the Institutional Review Board (IRB) IntegReview, 3815 S. Capital of Texas Hwy, Suite 320, Austin, TX 78704, Phone: 512-326-3001, Fax: 512-697-0085, http://www.integreview.com.

All research was performed in accordance with relevant guidelines and regulations; informed consent was obtained from all participants.

## Supplementary Information

Supplementary Information.

## Data Availability

The datasets generated and analyzed for the current study are available in the National Center for Biotechnology Information (NCBI) repository, Accession Number PRJNA882649.

## Abbreviations

KEGG: Kyoto encyclopedia of genes and genomes
NRPS: Nonribosomal peptide synthetase
PCA: Principal component analysis
PCoA: Principal coordinate analysis
SCFAs: Short-chain fatty acids

## References

[1] Vyas U, Ranganathan N (2012). Probiotics prebiotics, and synbiotics: Gut and beyond. Gastroenterol. Res. Pract..

[2] NIH Human Microbiome Portfolio Analysis Team (2019). A review of 10 years of human microbiome research activities at the US national institutes of health, fiscal years 2007–2016. Microbiome.

[3] Singh RK, Chang H-W, Yan D, Lee KM, Ucmak D, Wong K, Abrouk M, Farahnik B, Nakamura M, Zhu TH, Bhutani T, Liao W (2017). Influence of diet on the gut microbiome and implications for human health. J. Transl. Med..

[4] Tomova A, Bukovsky I, Rembert E, Yonas W, Alwarith J, Barnard ND, Kahleova H (2019). The effects of vegetarian and vegan diets on gut microbiota. Front. Nutr..

[5] Pezzuto JM (2016). Grapes and Health.

[6] Dave A, Park E-J, Kumar A, Parande F, Beyoğlu D, Idle JR, Pezzuto JM (2022). Consumption of Grapes modulates gene expression, reduces non-alcoholic fatty liver disease, and extends longevity in female C57BL/6J mice provided with a high-fat western-pattern diet. Foods..

[7] Parande F, Dave A, Park E-J, McAllister C, Pezzuto JM (2022). Effect of dietary grapes on female C57BL6/J mice consuming a high-fat diet: Behavioral and genetic changes. Antioxidants..

[8] Pezzuto JM (2019). Resveratrol: Twenty years of growth, development and controversy. Biomol. Ther..

[9] Pezzuto JM (2008). Grapes and human health: A perspective. J. Agric. Food Chem..

[10] Zhou L, Wang W, Huang J, Ding Y, Pan Z, Zhao Y, Zhang R, Hu B, Zeng X (2016). In vitro extraction and fermentation of polyphenols from grape seeds (*Vitis vinifera*) by human intestinal microbiota. Food Funct..

[11] Collins B, Hoffman J, Martinez K, Grace M, Lila MA, Cockrell C, Nadimpalli A, Chang E, Chuang C-C, Zhong W, Mackert J, Shen W, Cooney P, Hopkins R, McIntosh M (2016). A Polyphenol-rich fraction obtained from table grapes decreases adiposity, insulin resistance and markers of inflammation and impacts gut microbiota in high-fat-fed mice. J. Nutr. Biochem..

[12] Beyoğlu D, Park E-J, Quiñones-Lombraña A, Dave A, Parande F, Pezzuto JM, Idle JR (2022). Addition of grapes to both a standard and a high-fat western pattern diet modifies hepatic and urinary metabolite profiles in the mouse. Food Funct..

[13] Wang M, Keogh A, Treves S, Idle JR, Beyoğlu D (2016). The metabolomic profile of gamma-irradiated human hepatoma and muscle cells reveals metabolic changes consistent with the warburg effect. PeerJ.

[14] Simillion C, Semmo N, Idle JR, Beyoğlu D (2017). Robust regression analysis of GCMS data reveals differential rewiring of metabolic networks in hepatitis B and C patients. Metabolites.

[15] Yang J, Kurnia P, Henning SM, Lee R, Huang J, Garcia MC, Surampudi V, Heber D, Li Z (2021). Effect of standardized grape powder consumption on the gut microbiome of healthy subjects: A pilot study. Nutrients.

[16] Barrett HL, Gomez-Arango LF, Wilkinson SA, McIntyre HD, Callaway LK, Morrison M, Dekker Nitert M (2018). A vegetarian diet is a major determinant of gut microbiota composition in early pregnancy. Nutrients.

[17] Shao M, Zhu Y (2020). Long-term metal exposure changes gut microbiota of residents surrounding a mining and smelting area. Sci. Rep..

[18] Chen W, Liu F, Ling Z, Tong X, Xiang C (2012). Human intestinal lumen and mucosa-associated microbiota in patients with colorectal cancer. PLoS ONE.

[19] Tuovinen E, Keto J, Nikkilä J, Mättö J, Lähteenmäki K (2013). Cytokine response of human mononuclear cells induced by intestinal *Clostridium* species. Anaerobe.

[20] Martinović A, Cocuzzi R, Arioli S, Mora D (2020). *Streptococcus thermophilus*: To survive, or not to survive the gastrointestinal tract, that is the question!. Nutrients.

[21] Haas KN, Blanchard JL (2020). Reclassification of the *Clostridium clostridioforme* and *Clostridium sphenoides* clades as *Enterocloster* gen. nov. and *Lacrimispora* gen. nov., Including Reclassification of 15 Taxa. Int. J. Syst. Evol. Microbiol..

[22] Benítez-Páez A, Gómez Del Pugar EM, López-Almela I, Moya-Pérez Á, Codoñer-Franch P, Sanz Y (2020). Depletion of *Blautia* species in the microbiota of obese children relates to intestinal inflammation and metabolic phenotype worsening. MSystems..

[23] Markowiak-Kopeć P, Śliżewska K (2020). The effect of probiotics on the production of short-chain fatty acids by human intestinal microbiome. Nutrients.

[24] Cheng SL, Li X, Lehmler H-J, Phillips B, Shen D, Cui JY (2018). Gut microbiota modulates interactions between polychlorinated biphenyls and bile acid homeostasis. Toxicol. Sci..

[25] Deaver JA, Eum SY, Toborek M (2018). Circadian disruption changes gut microbiome taxa and functional gene composition. Front. Microbiol..

[26] Kropp C, Le Corf K, Relizani K, Tambosco K, Martinez C, Chain F, Rawadi G, Langella P, Claus SP, Martin R (2021). The keystone commensal bacterium *Christensenella minuta* DSM 22607 displays anti-inflammatory properties both in vitro and in vivo. Sci. Rep..

[27] Bui TPN, Schols HA, Jonathan M, Stams AJM, de Vos WM, Plugge CM (2019). Mutual metabolic interactions in co-cultures of the intestinal anaerostipes rhamnosivorans with an acetogen methanogen, or pectin-degrader affecting butyrate production. Front. Microbiol..

[28] Rivière A, Selak M, Lantin D, Leroy F, De Vuyst L (2016). Bifidobacteria and butyrate-producing colon bacteria: Importance and strategies for their stimulation in the human gut. Front. Microbiol..

[29] Takeo M, Nishimura M, Shirai M, Takahashi H, Negoro S (2007). Purification and characterization of catechol 2,3-dioxygenase from the aniline degradation pathway of *Acinetobacter* sp. YAA and its mutant enzyme, which resists substrate inhibition. Biosci. Biotechnol. Biochem..

[30] Goodman MF (2002). Error-prone repair DNA Polymerases in prokaryotes and eukaryotes. Annu. Rev. Biochem..

[31] Verma S, Dixit R, Pandey KC (2016). Cysteine proteases: Modes of activation and future prospects as pharmacological targets. Front. Pharmacol..

[32] Vasiliou V, Vasiliou K, Nebert DW (2009). Human ATP-binding cassette (ABC) transporter family. Hum. Genomics..

[33] Shattuck-Eidens DM, Kadner RJ (1981). Exogenous induction of the *Escherichia coli* hexose phosphate transport system defined by uhp-lac operon fusions. J. Bacteriol..

[34] Kareem HM (2020). Oxidoreductases: Significance for humans and microorganism. IntechOpen.

[35] Süssmuth R, Müller J, von Döhren H, Molnár I (2010). Fungal cyclooligomer depsipeptides: From classical biochemistry to combinatorial biosynthesis. Nat. Prod. Rep..

[36] Santos LO, Garcia-Gomes AS, Catanho M, Sodre CL, Santos ALS, Branquinha MH, d’Avila-Levy CM (2013). Aspartic peptidases of human pathogenic trypanosomatids: perspectives and trends for chemotherapy. Curr. Med. Chem..

[37] Frigaard N-U, Hatti-Kaul R, Mamo G, Mattiasson B (2016). Biotechnology of anoxygenic phototrophic bacteria. Anaerobes in Biotechnology.

[38] Huang N, Hua D, Zhan G, Li S, Zhu B, Jiang R, Yang L, Bi J, Xu H, Hashimoto K, Luo A, Yang C (2019). Role of actinobacteria and coriobacteriia in the antidepressant effects of ketamine in an inflammation model of depression. Pharmacol. Biochem. Behav..

[39] Gupta RS, Nanda A, Khadka B (2017). Novel molecular, structural and evolutionary characteristics of the phosphoketolases from bifidobacteria and *Coriobacteriales*. PLoS ONE.

[40] Liu H, Zhang H, Wang X, Yu X, Hu C, Zhang X (2018). The family *Coriobacteriaceae* is a potential contributor to the beneficial effects of roux-en-Y gastric bypass on type 2 diabetes. Surg. Obes. Relat. Dis..

[41] Chen J, Wright K, Davis JM, Jeraldo P, Marietta EV, Murray J, Nelson H, Matteson EL, Taneja V (2016). An expansion of rare lineage intestinal microbes characterizes rheumatoid arthritis. Genome Med..

[42] Bag S, Ghosh TS, Das B (2017). Complete genome sequence of *Collinsella aerofaciens* isolated from the gut of a healthy indian subject. Genome Announc..

[43] Zhang X, Zhong H, Li Y, Shi Z, Ren H, Zhang Z, Zhou X, Tang S, Han X, Lin Y, Yang F, Wang D, Fang C, Fu Z, Wang L, Zhu S, Hou Y, Xu X, Yang H, Wang J, Kristiansen K, Li J, Ji L (2021). Sex- and age-related trajectories of the adult human gut microbiota shared across populations of different ethnicities. Nat. Aging..

[44] Laitinen K, Mokkala K (2019). Overall dietary quality relates to gut microbiota diversity and abundance. Int. J. Mol. Sci..

[45] Chuang C-C, Shen W, Chen H, Xie G, Jia W, Chung S, McIntosh MK (2012). Differential effects of grape powder and its extract on glucose tolerance and chronic inflammation in high-fat-fed obese mice. J. Agric. Food Chem..

[46] Chuang C-C, McIntosh MK (2011). Potential mechanisms by which polyphenol-rich grapes prevent obesity-mediated inflammation and metabolic diseases. Annu. Rev. Nutr..

[47] Thompson PS (2020). New insights into abasic site repair and tolerance. DNA Repair.

[48] Zheng Y, Sheppard TL (2004). Half-life and dna strand scission products of 2-deoxyribonolactone oxidative DNA damage lesions. Chem. Res. Toxicol..

[49] Pezzuto JM, Dave A, Park E-J, Beyoğlu D, Idle JR (2022). Short-term grape consumption diminishes UV-induced skin erythema. Antioxidants..

[50] Djurdjevic I, Zelder O, Buckel W (2011). Production of glutaconic acid in a recombinant *Escherichia coli* strain. Appl. Environ. Microbiol..

[51] Zhou Q, Deng J, Pan X, Meng D, Zhu Y, Bai Y, Shi C, Duan Y, Wang T, Li X, Sluijter JP, Xiao J (2022). Gut microbiome mediates the protective effects of exercise after myocardial infarction. Microbiome..

[52] Xiong X, Liu D, Wang Y, Zeng T, Peng Y (2016). Urinary 3-(3-hydroxyphenyl)-3-hydroxypropionic acid, 3-hydroxyphenylacetic acid, and 3-hydroxyhippuric acid are elevated in children with autism spectrum disorders. Biomed. Res. Int..

[53] Martín M, Gibello A, Fernández J, Ferrer E, Garrido-Pertierra A (1991). Catabolism of 3- and 4-hydroxyphenylacetic acid by *Klebsiella pneumoniae*. J. Gen. Microbiol..

[54] van Breemen RB, Wright B, Li Y, Nosal D, Burton T, Pezzuto JM (2016). Standardized grape powder for basic and clinical research. Grapes and Health.

[55] Webb-Robertson B-J, Kim Y-M, Zink EM, Hallaian KA, Zhang Q, Madupu R, Waters KM, Metz TO (2014). A statistical analysis of the effects of urease pre-treatment on the measurement of the urinary metabolome by gas chromatography-mass spectrometry. Metabolomics.

[56] McGough JJ, Faraone SV (2009). Estimating the size of treatment effects. Psychiatry.

