# Supplementary material for: Influence of grape consumption on the human microbiome
Source: Sci Rep. 2023 May 12;13:7706. doi: 10.1038/s41598-023-34813-5 (PMC10182090; doi:10.1038/s41598-023-34813-5)
Supplement: Supplementary file 1 — Supplementary Information. [file 41598_2023_34813_MOESM1_ESM.pdf]

Supplement 1. Alpha-diversity of the subject population and subgroups.

A

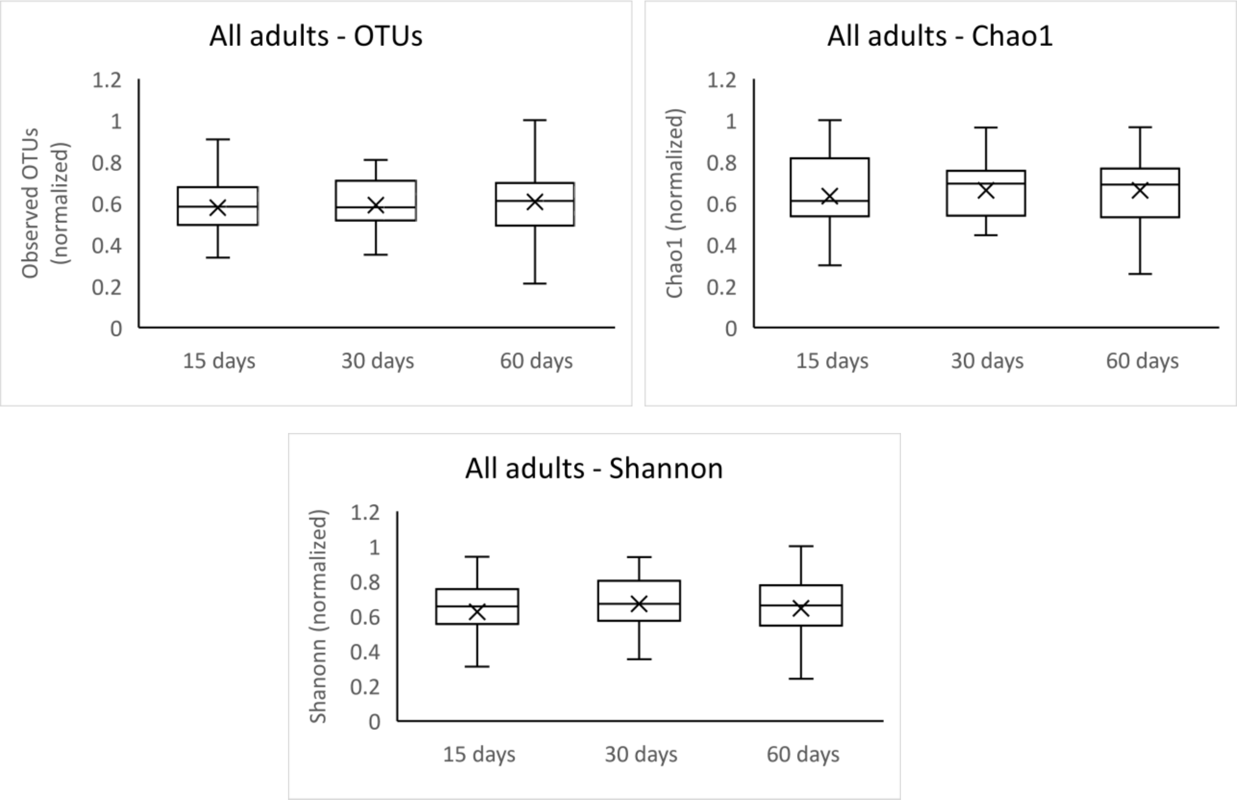

B

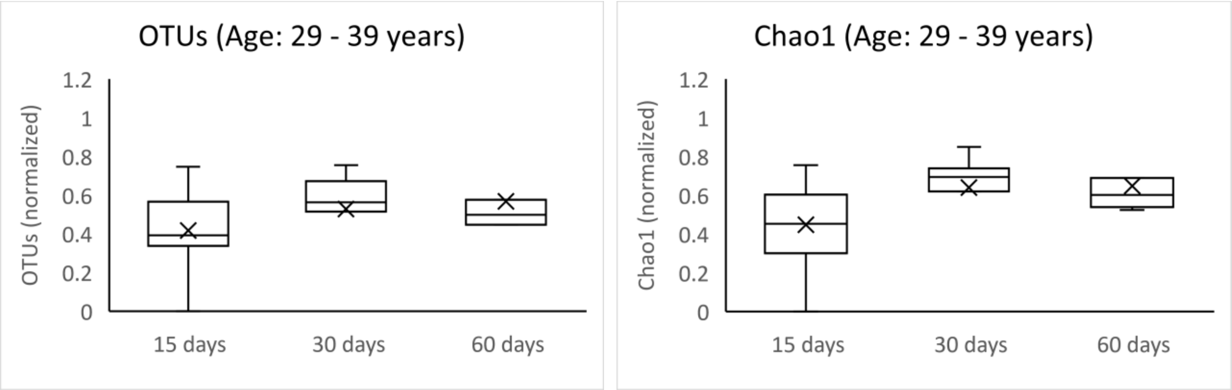

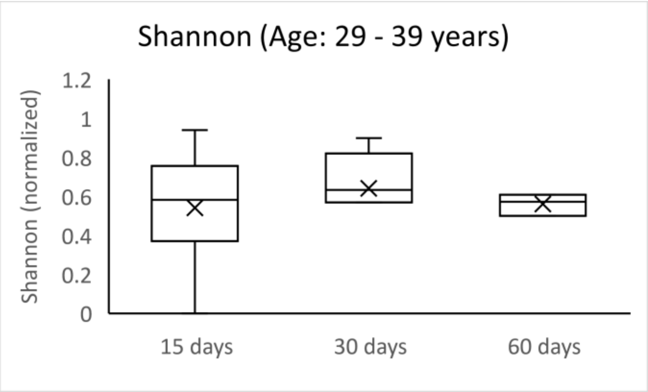
Shannon (Age: 29 - 39 years)
Shannon (normalized)
1.2
1
0.8
0.6
0.4
0.2
0
15 days
30 days
60 days

C

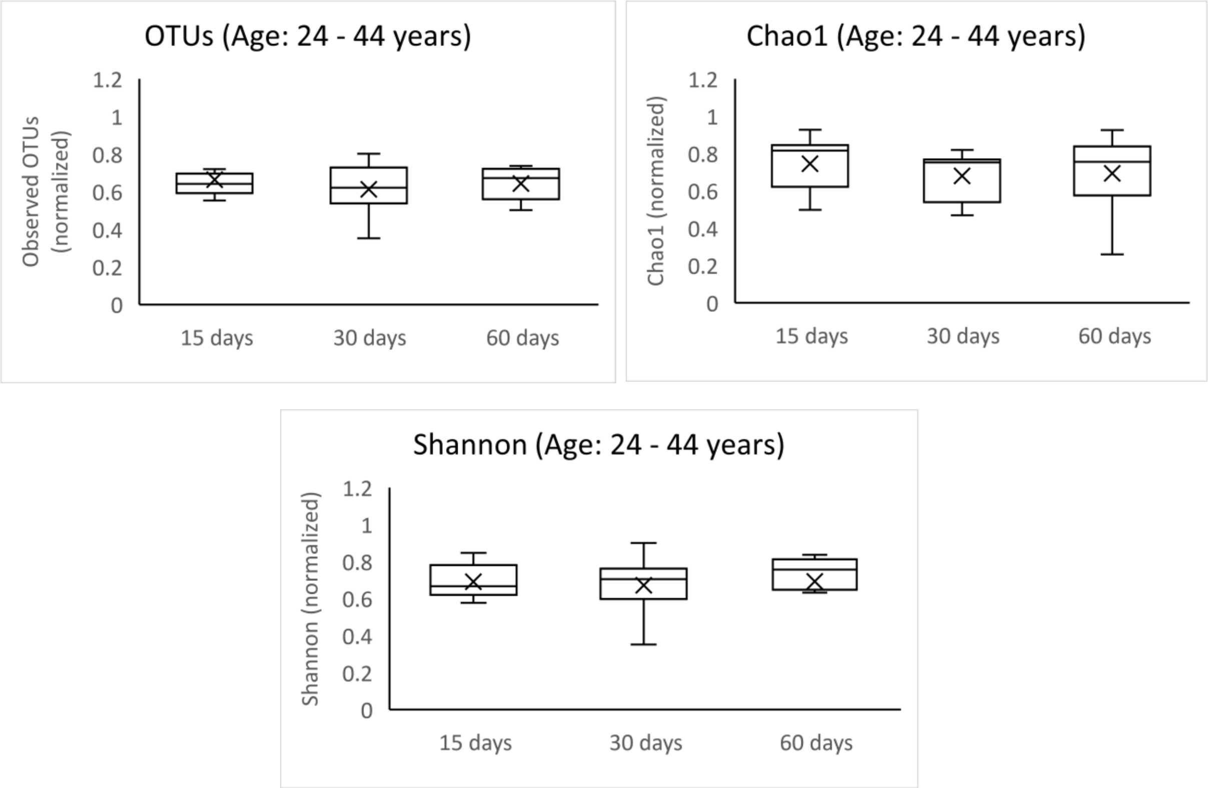
OTUs (Age: 24 - 44 years)
Observed OTUs (normalized)
1.2
1
0.8
0.6
0.4
0.2
0
15 days
30 days
60 days
Chao1 (Age: 24 - 44 years)
Chao1 (normalized)
1.2
1
0.8
0.6
0.4
0.2
0
15 days
30 days
60 days
Shannon (Age: 24 - 44 years)
Shannon (normalized)
1.2
1
0.8
0.6
0.4
0.2
0
15 days
30 days
60 days

D

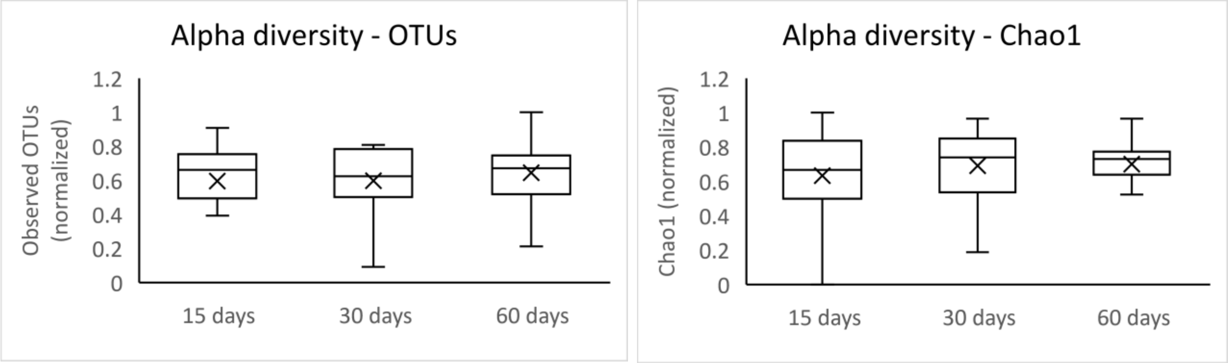
Alpha diversity - OTUs
Observed OTUs (normalized)
1.2
1
0.8
0.6
0.4
0.2
0
15 days
30 days
60 days
Alpha diversity - Chao1
Chao1 (normalized)
1.2
1
0.8
0.6
0.4
0.2
0
15 days
30 days
60 days

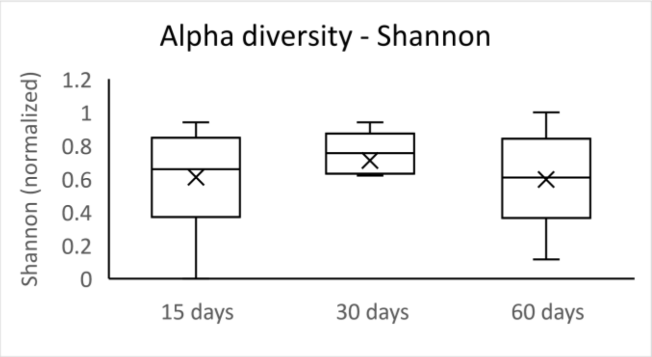

Figure S1. Effect of 15-days of grape powder intake on gut microbiota and followed by 30 days of washout period. OTU, Chao1 and Shannon indices were measured for **A.** All the adults, *n* = 29. **B.** Females from age 29 to 39, *n* = 7. **C.** Males from 24 to 44 age, *n* = 7. **D.** Females and males selected based on unique microbiome profiles over the three time periods, *n* = 11.

Supplement 2. Beta-diversity of subject population and subgroups.

A (i)

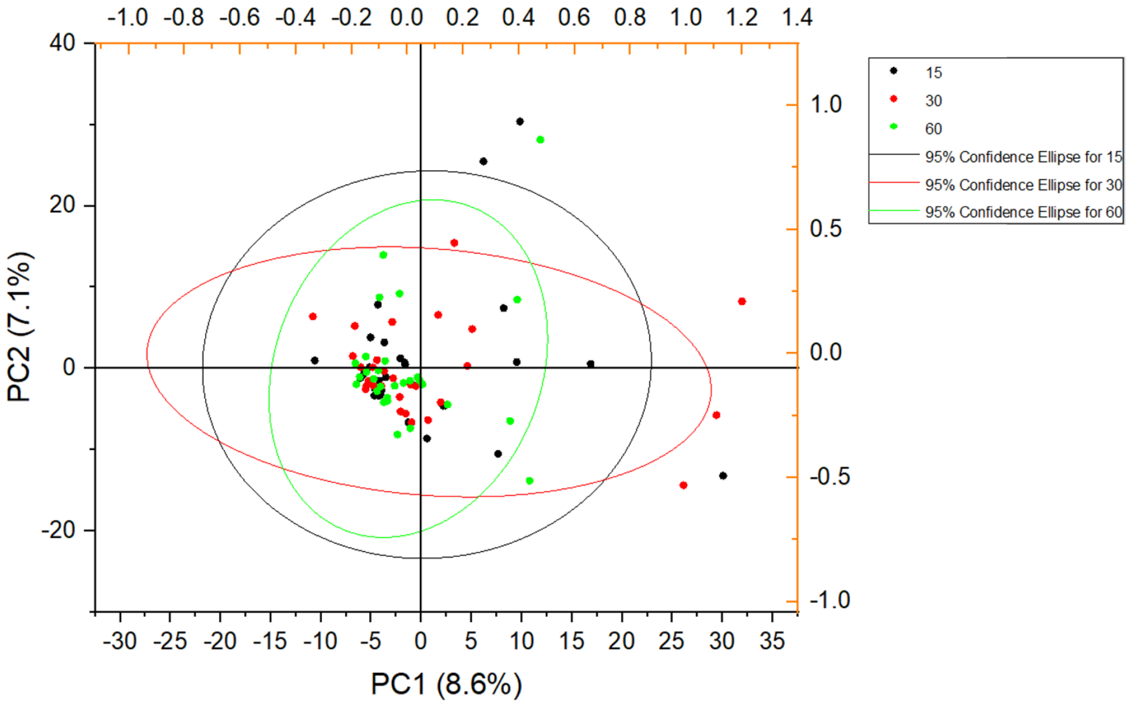

A (ii)

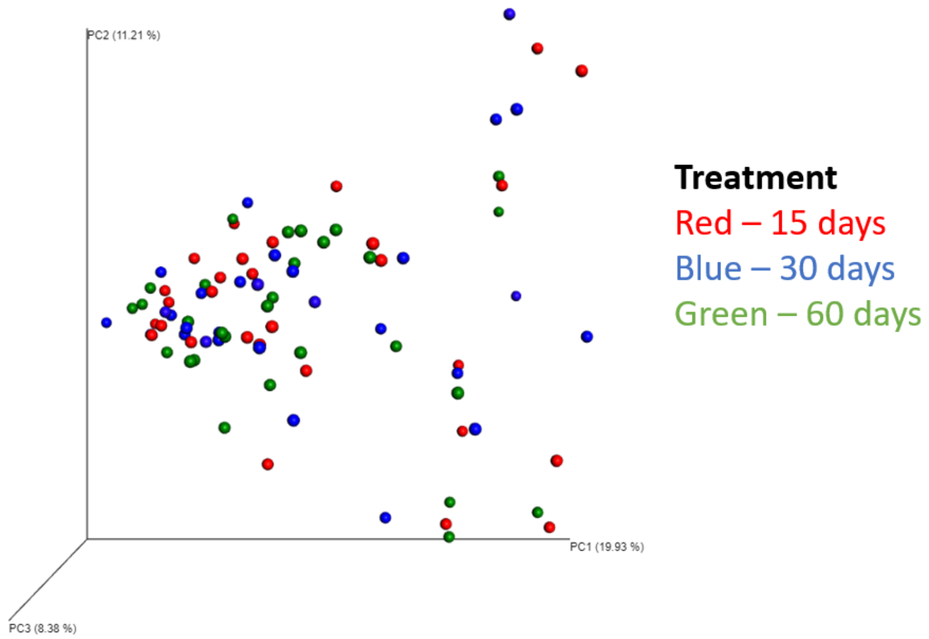
PC2 (11.21 %)
PC1 (19.93 %)
PC3 (8.38 %)
Treatment
Red – 15 days
Blue – 30 days
Green – 60 days

A (iii)

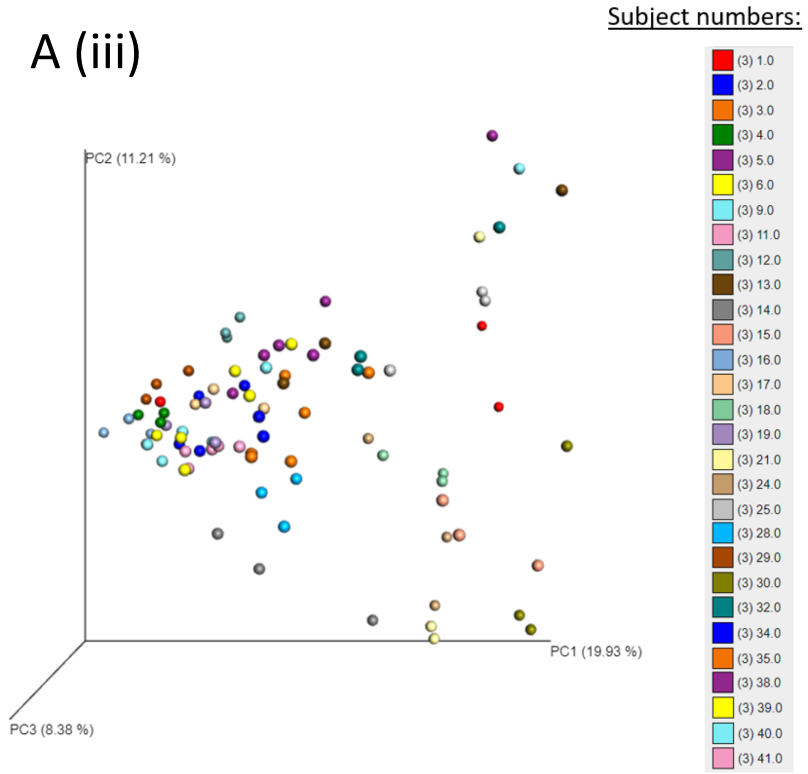
Subject numbers:
(3) 1.0
(3) 2.0
(3) 3.0
(3) 4.0
(3) 5.0
(3) 6.0
(3) 9.0
(3) 11.0
(3) 12.0
(3) 13.0
(3) 14.0
(3) 15.0
(3) 16.0
(3) 17.0
(3) 18.0
(3) 19.0
(3) 21.0
(3) 24.0
(3) 25.0
(3) 28.0
(3) 29.0
(3) 30.0
(3) 32.0
(3) 34.0
(3) 35.0
(3) 38.0
(3) 39.0
(3) 40.0
(3) 41.0
PC2 (11.21 %)
PC1 (19.93 %)
PC3 (8.38 %)

B (i)

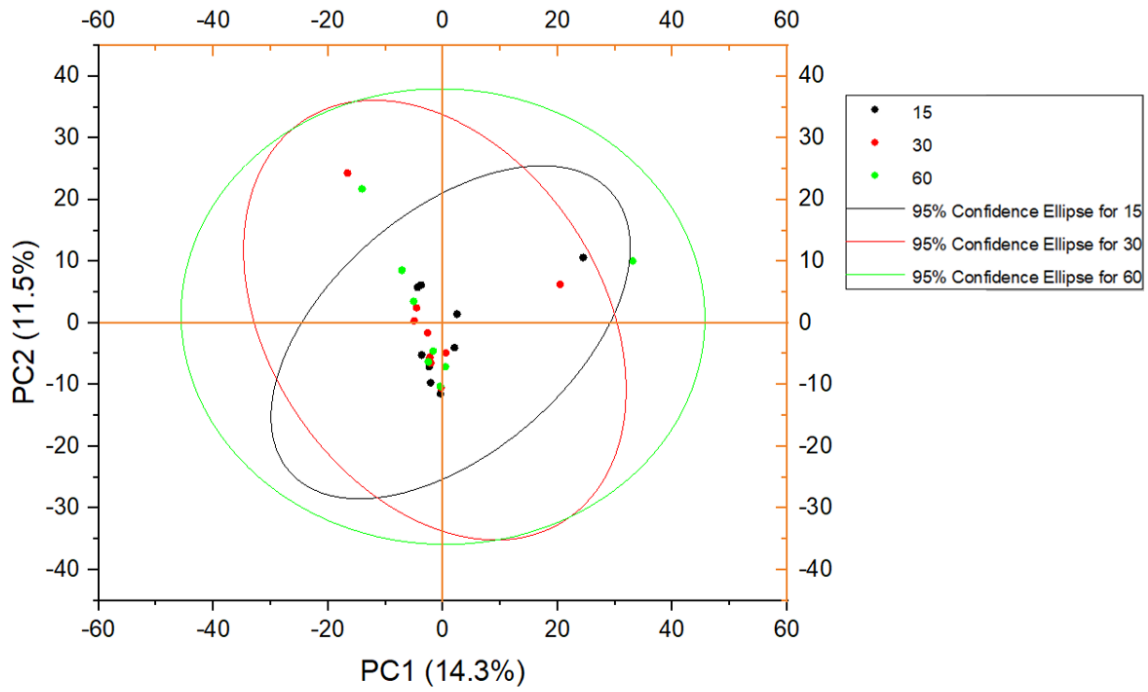

PC1 (14.3%)
PC2 (11.5%)
15
30
60
95% Confidence Ellipse for 15
95% Confidence Ellipse for 30
95% Confidence Ellipse for 60

B (ii)

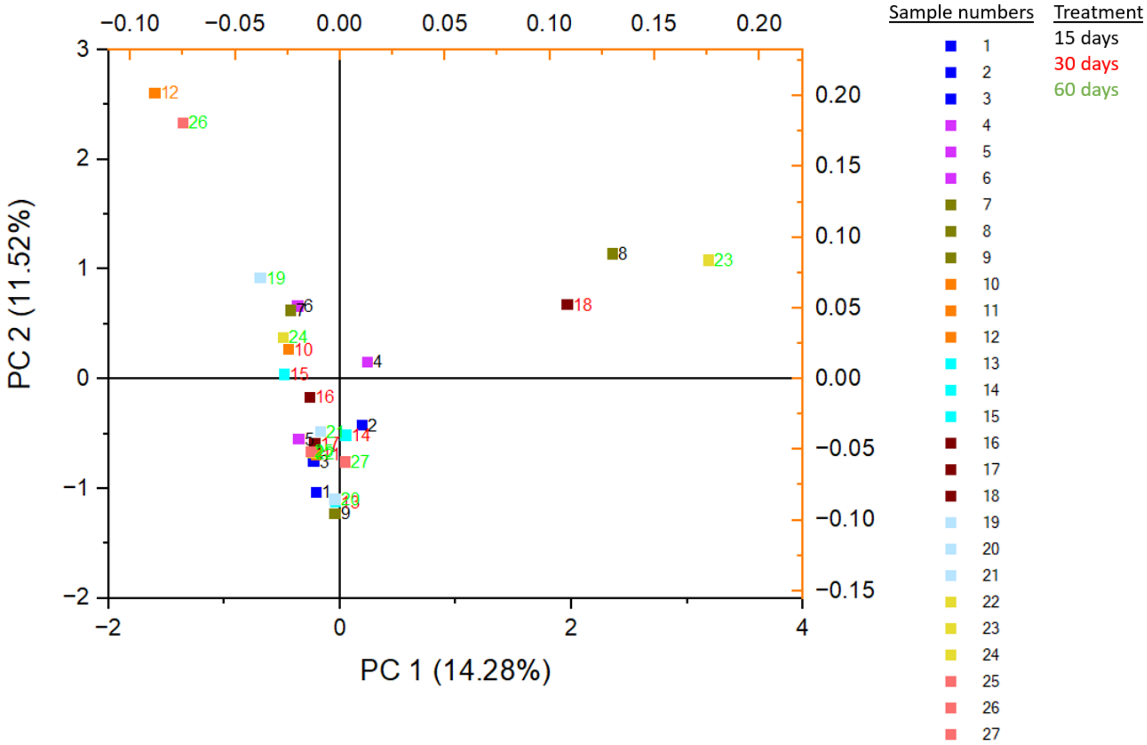

PC 1 (14.28%)
PC 2 (11.52%)
Sample numbers
Treatment
15 days
30 days
60 days

B (iii)

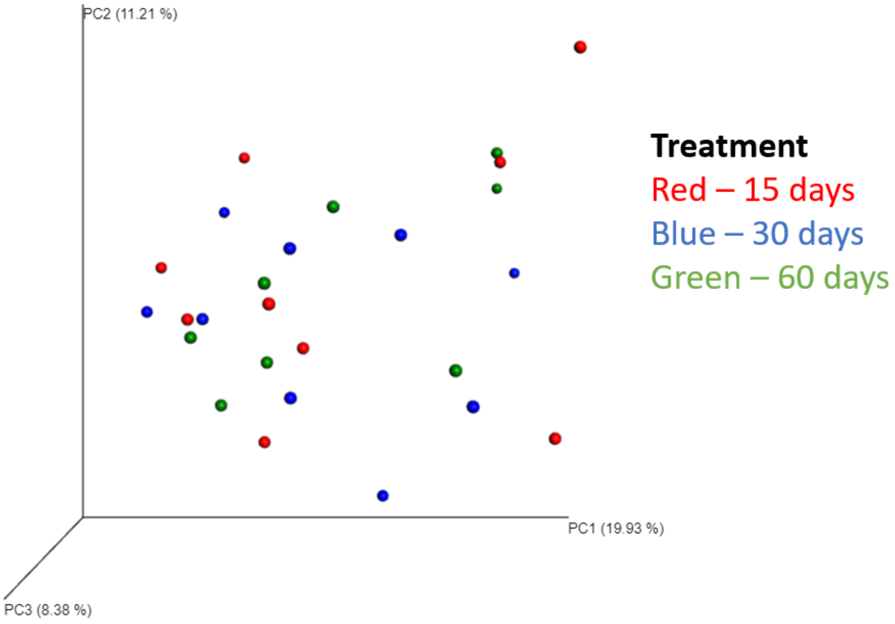
PC2 (11.21 %)
PC1 (19.93 %)
PC3 (8.38 %)
Treatment
Red – 15 days
Blue – 30 days
Green – 60 days

B (iv)

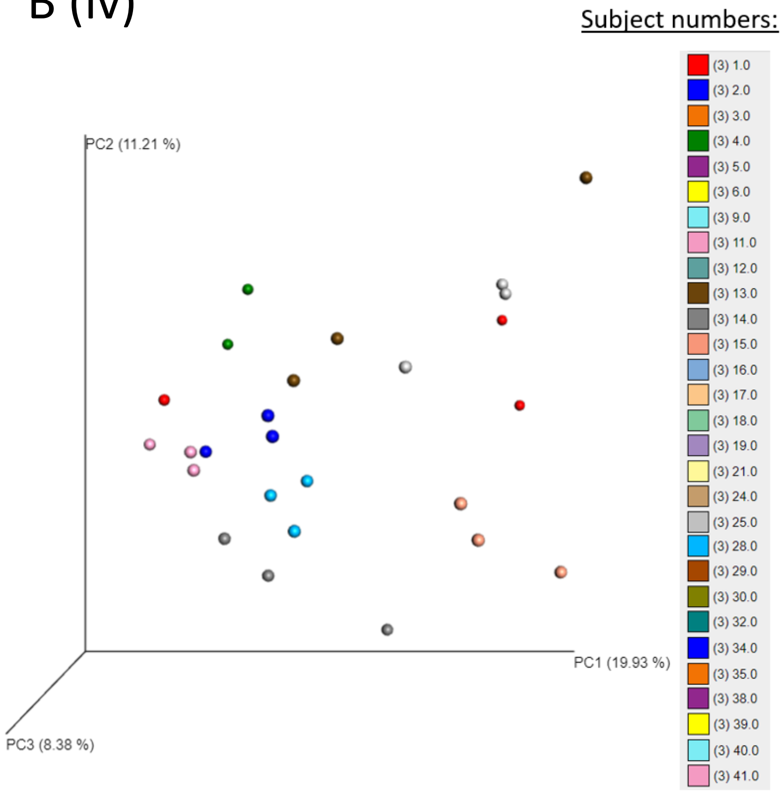
Subject numbers:
(3) 1.0
(3) 2.0
(3) 3.0
(3) 4.0
(3) 5.0
(3) 6.0
(3) 9.0
(3) 11.0
(3) 12.0
(3) 13.0
(3) 14.0
(3) 15.0
(3) 16.0
(3) 17.0
(3) 18.0
(3) 19.0
(3) 21.0
(3) 24.0
(3) 25.0
(3) 28.0
(3) 29.0
(3) 30.0
(3) 32.0
(3) 34.0
(3) 35.0
(3) 38.0
(3) 39.0
(3) 40.0
(3) 41.0
PC2 (11.21 %)
PC1 (19.93 %)
PC3 (8.38 %)

C (i)

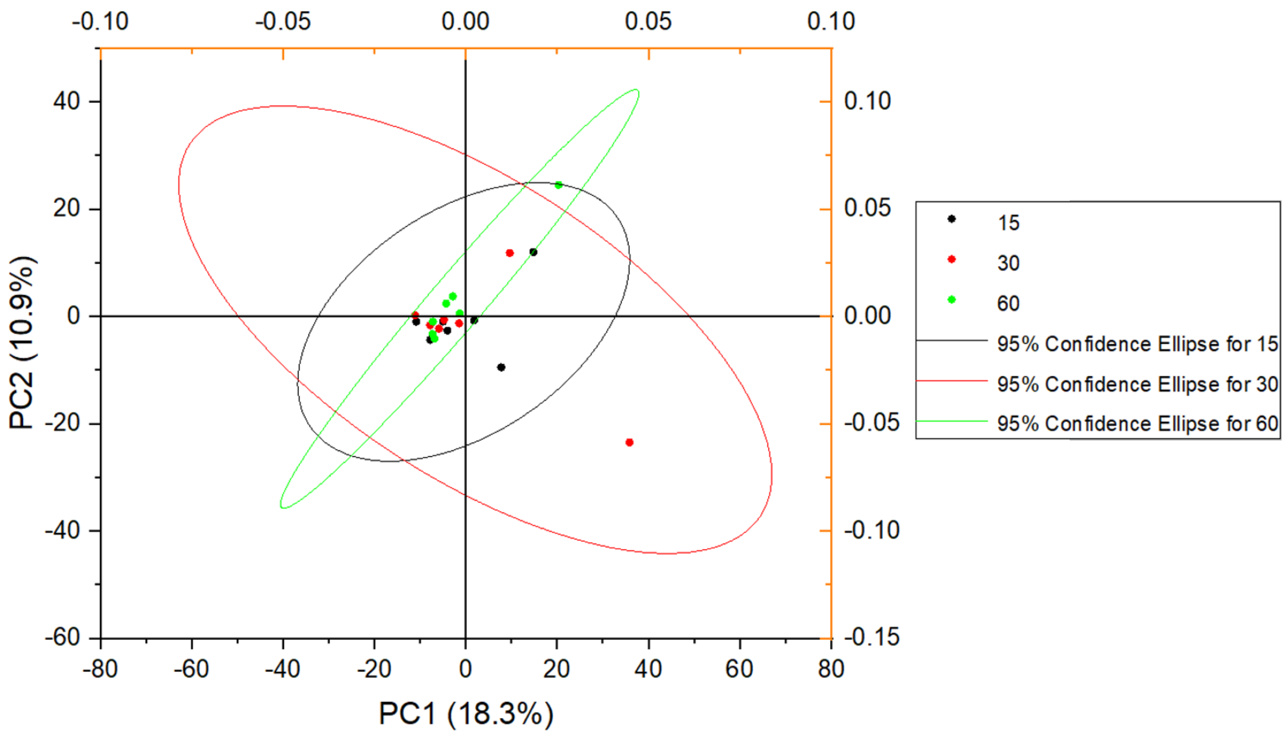

PC1 (18.3%)
PC2 (10.9%)
15
30
60
95% Confidence Ellipse for 15
95% Confidence Ellipse for 30
95% Confidence Ellipse for 60

C (ii)

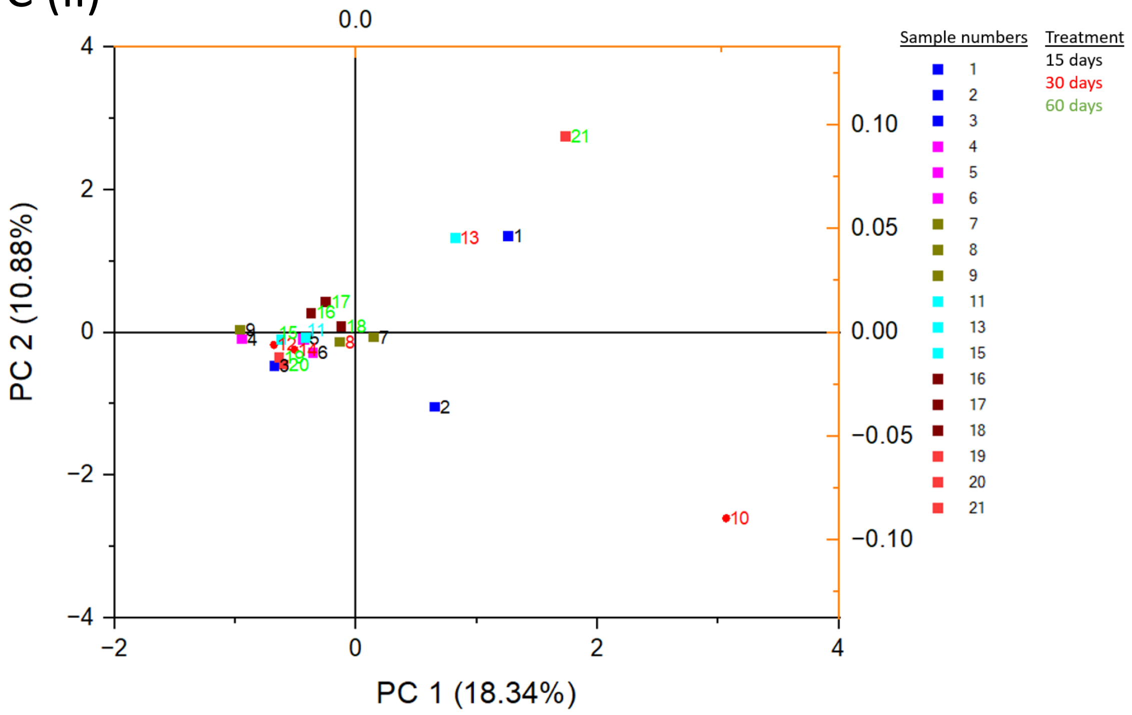

PC 1 (18.34%)
PC 2 (10.88%)
Sample numbers
Treatment
15 days
30 days
60 days
1
2
3
4
5
6
7
8
9
11
13
15
16
17
18
19
20
21

C (iii)

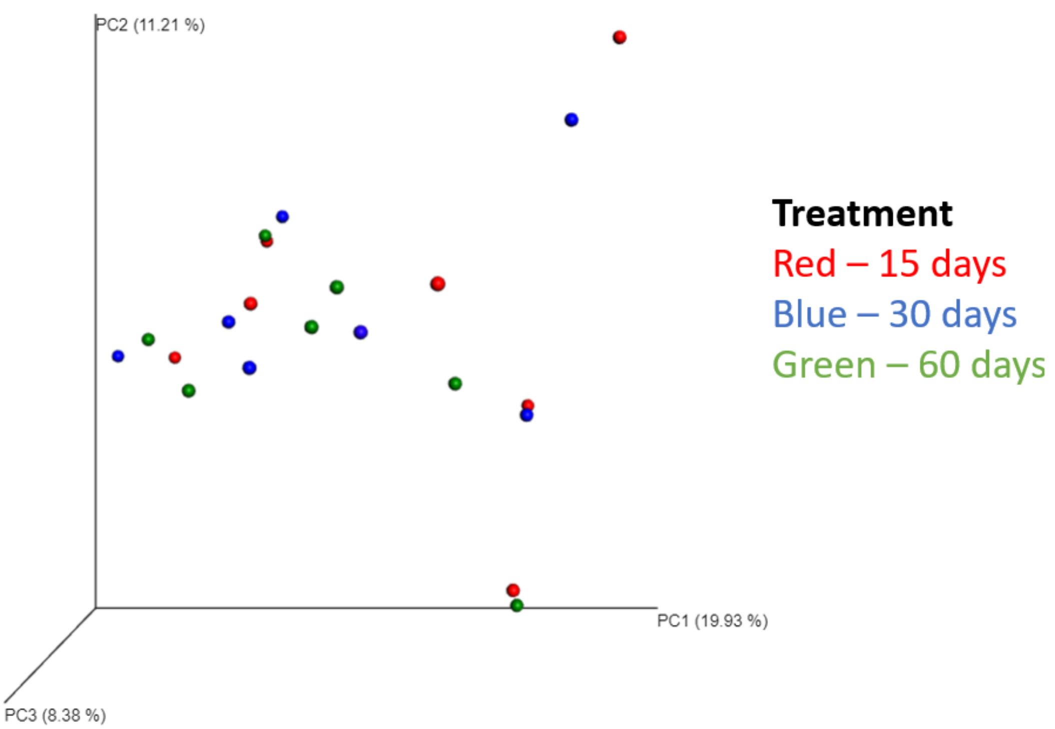
PC2 (11.21 %)
PC1 (19.93 %)
PC3 (8.38 %)
Treatment
Red – 15 days
Blue – 30 days
Green – 60 days

C (iv)

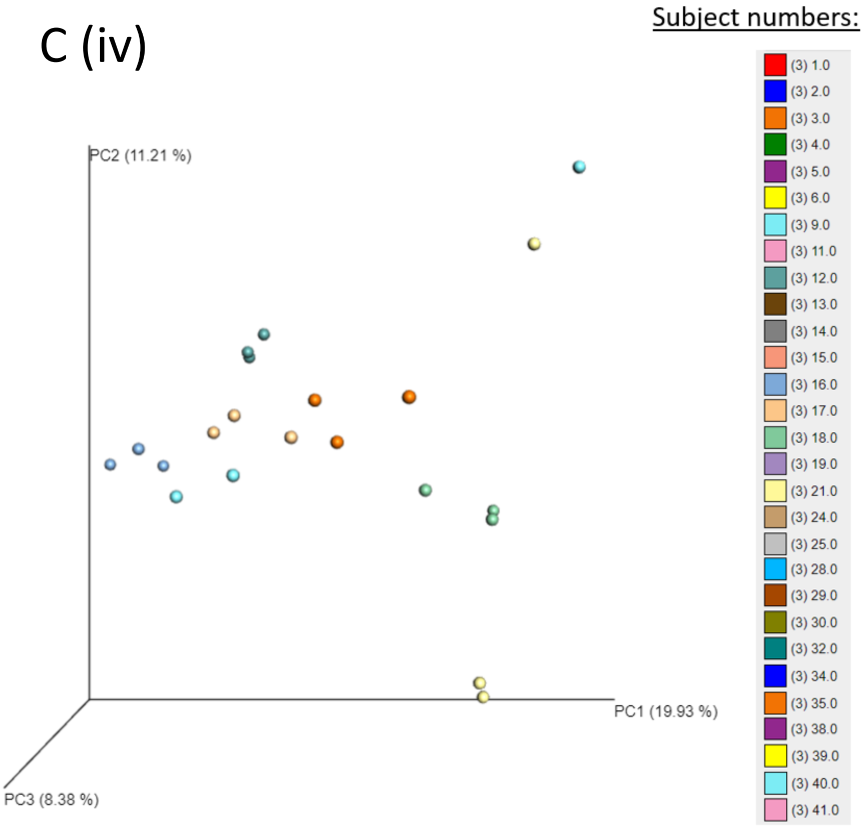
Subject numbers:
PC2 (11.21 %)
PC1 (19.93 %)
PC3 (8.38 %)
(3) 1.0
(3) 2.0
(3) 3.0
(3) 4.0
(3) 5.0
(3) 6.0
(3) 9.0
(3) 11.0
(3) 12.0
(3) 13.0
(3) 14.0
(3) 15.0
(3) 16.0
(3) 17.0
(3) 18.0
(3) 19.0
(3) 21.0
(3) 24.0
(3) 25.0
(3) 28.0
(3) 29.0
(3) 30.0
(3) 32.0
(3) 34.0
(3) 35.0
(3) 38.0
(3) 39.0
(3) 40.0
(3) 41.0

D (i)

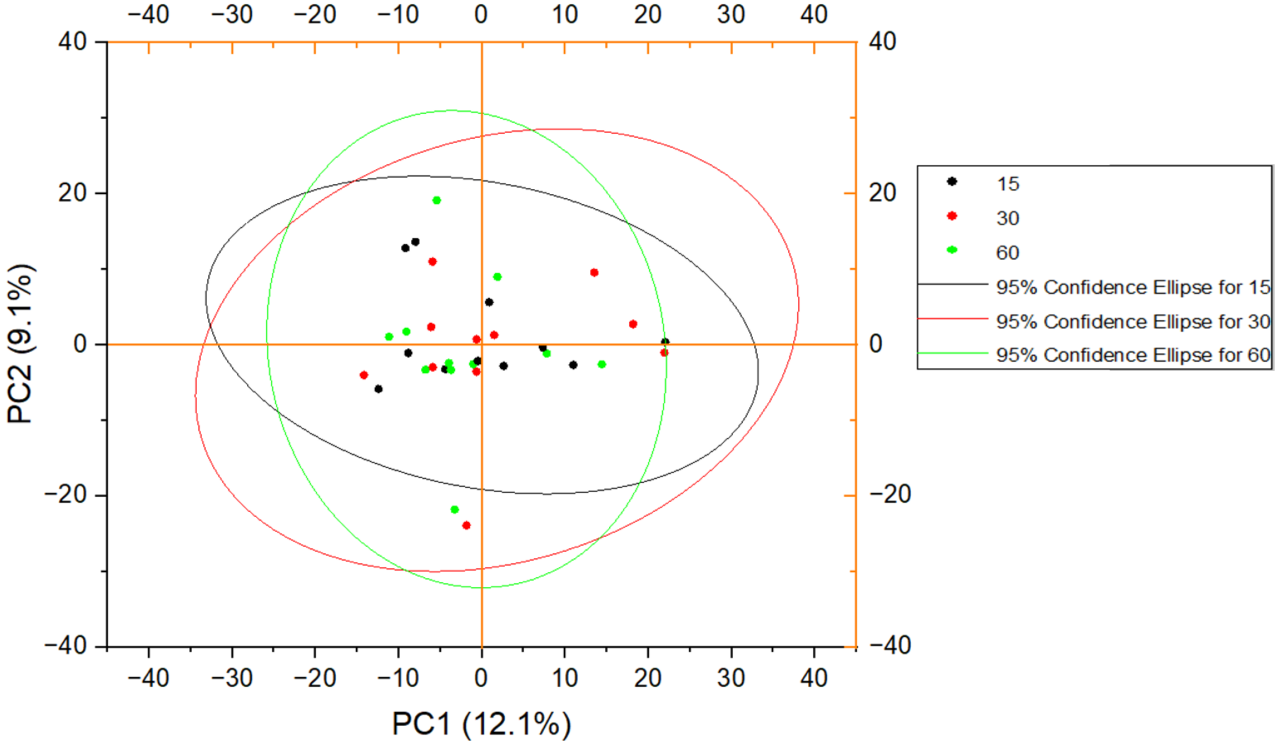
PC1 (12.1%)
PC2 (9.1%)
15
30
60
95% Confidence Ellipse for 15
95% Confidence Ellipse for 30
95% Confidence Ellipse for 60

D (ii)

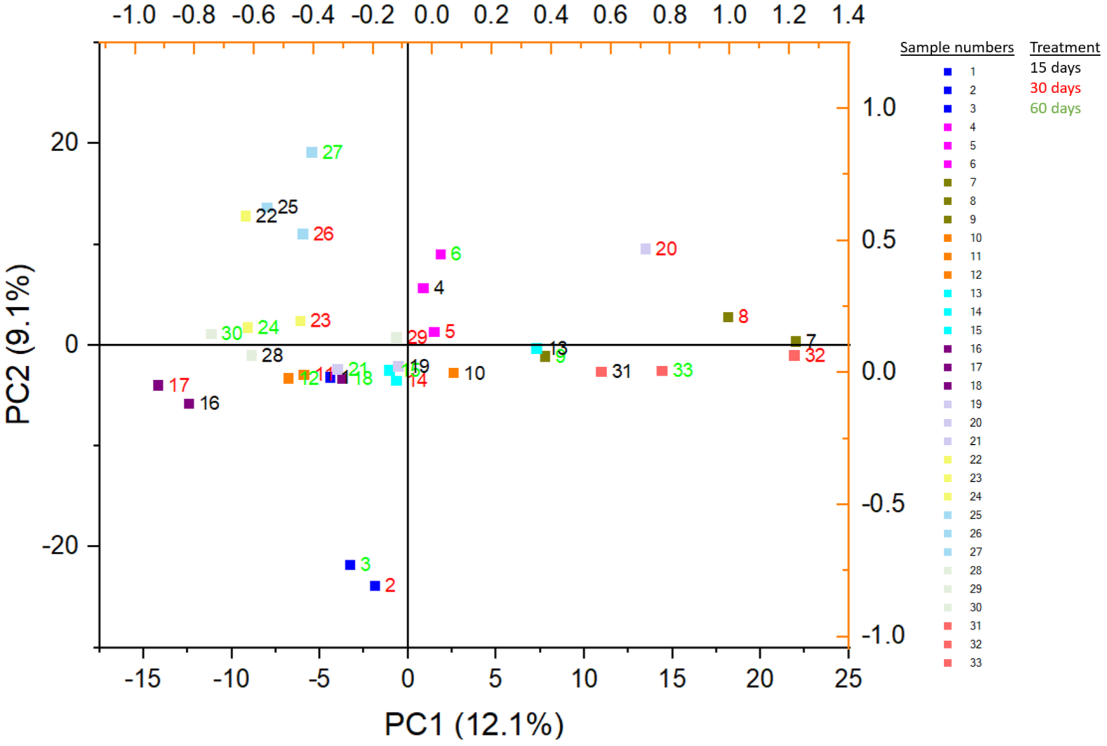
PC1 (12.1%)
PC2 (9.1%)
Sample numbers
Treatment
15 days
30 days
60 days

D (iii)

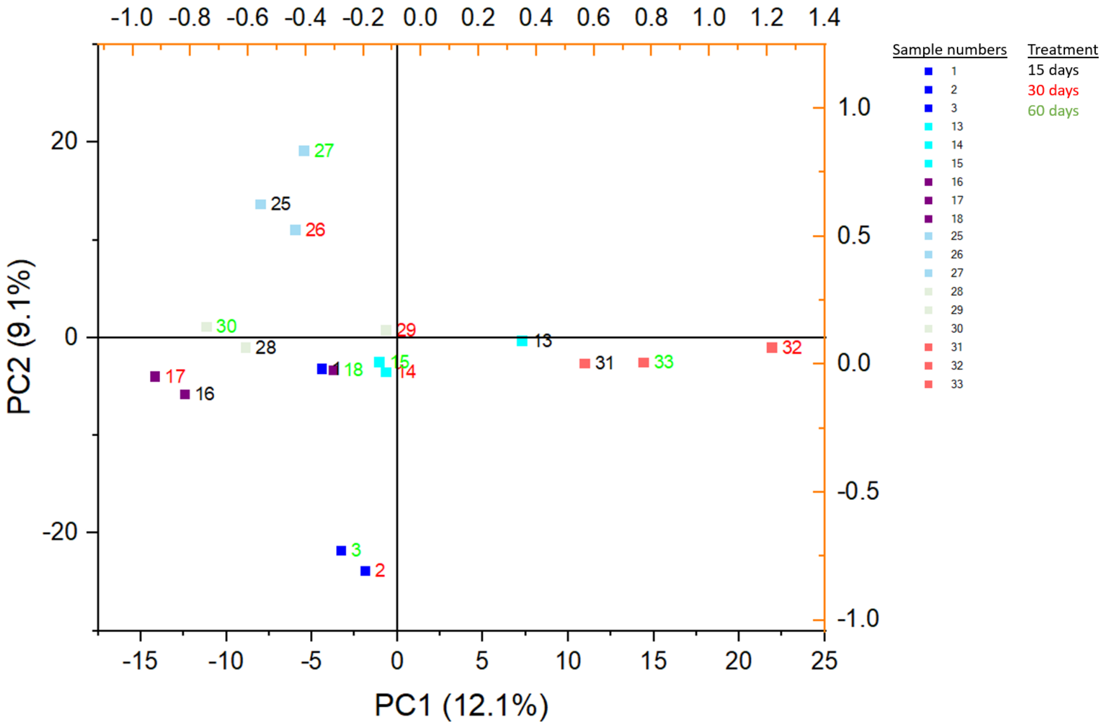
PC1 (12.1%)
PC2 (9.1%)
Sample numbers
1
2
3
13
14
15
16
17
18
25
26
27
28
29
30
31
32
33
Treatment
15 days
30 days
60 days

D (iv)

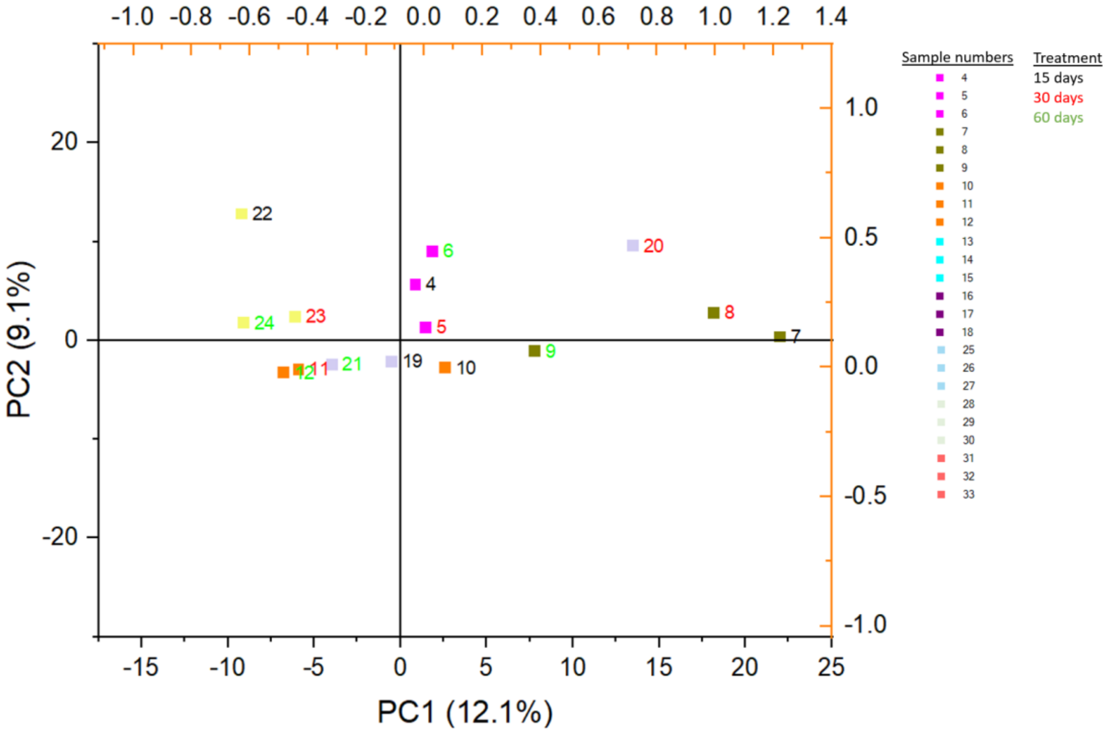
PC1 (12.1%)
PC2 (9.1%)
Sample numbers
4
5
6
7
8
9
10
11
12
13
14
15
16
17
18
25
26
27
28
29
30
31
32
33
Treatment
15 days
30 days
60 days

D (v)

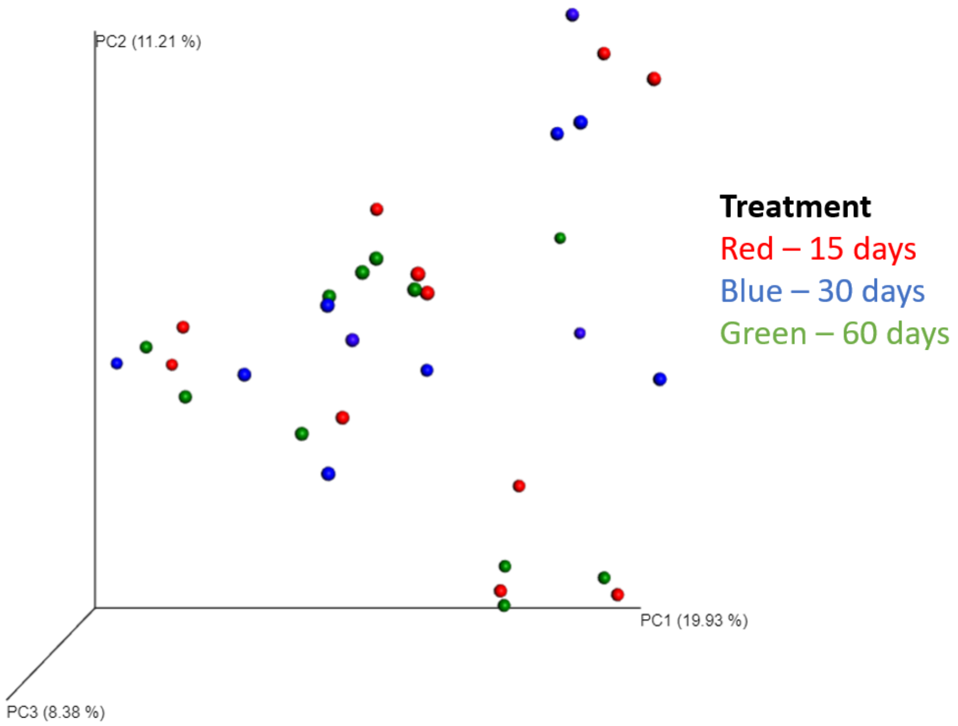
PC2 (11.21 %)
PC1 (19.93 %)
PC3 (8.38 %)
Treatment
Red – 15 days
Blue – 30 days
Green – 60 days

D (vi)

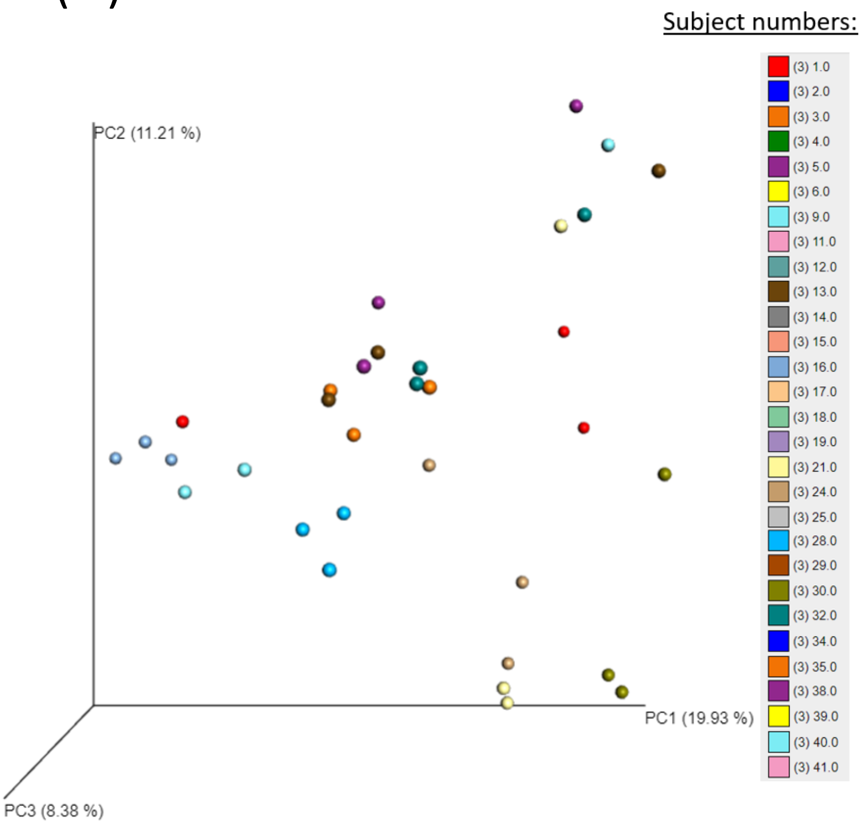
Subject numbers:
(3) 1.0
(3) 2.0
(3) 3.0
(3) 4.0
(3) 5.0
(3) 6.0
(3) 9.0
(3) 11.0
(3) 12.0
(3) 13.0
(3) 14.0
(3) 15.0
(3) 16.0
(3) 17.0
(3) 18.0
(3) 19.0
(3) 21.0
(3) 24.0
(3) 25.0
(3) 28.0
(3) 29.0
(3) 30.0
(3) 32.0
(3) 34.0
(3) 35.0
(3) 38.0
(3) 39.0
(3) 40.0
(3) 41.0
PC2 (11.21 %)
PC1 (19.93 %)
PC3 (8.38 %)

Figure S1. Beta-diversity determined by Bray–Curtis dissimilarity displayed by PCA and PCoA plots. Each subject is represented by a dot. The timeline is as follows: 15 day baseline measurement, 15 days of grape diet (represented by 30 days), and a 30 day washout period (represented by 60 days) **A (i).** PCA plot along with the cluster (95% confidence interval) shows the data for all the samples taken together. **A (ii)** PCoA plot for all the samples showing different stages of diet. **A (iii)** PCoA plots for all the samples showing the transition in each subject**. B.** Males from age 24 to 44, *n* = 9. **B (i).** PCA plot with 95% confidence interval cluster. **B (ii).** PCA analysis for the individual subjects. **B (iii).** PCoA plot showing different stages of diet. **B (iv).** PCoA plot for the transition in each subject through different stages of diet. **C.** Females from age 29 to 39, *n* = 7. **C (i).** PCA plot clustered with the 95% confidence interval for 15, 30 and 60 days. **C (ii).** PCA analysis for the individual subjects. **C (iii).** PCoA plot showing different stages of diet. **C (iv).** PCoA plot for the transition in each subject through different stages of diet. **D.** Females and males selected based on unique microbiome profiles over the three time periods, *n* = 11. **D (i).** PCA plot along with the cluster (95% confidence interval). **D (ii).** The plot shows the individual subjects on 15, 30 and 60 days. **D (iii)** The plot represents male subjects where, *n* = 5. Each color represents individual subject in the study. **D (iv).** The plot represents female subjects where *n* = 6. Each symbol represents individual subject in the study. **D (v).** PCoA plot showing different stages of diet. **B (vi).** PCoA plot for the transition in each subject through different stages of diet.

Supplement 3. Data obtained with select subjects based on unique differences observed in taxonomic profiles on Days 15, 30 and 60 (*n* = 11) and overlays of area charts representing diversity of species for all subjects (Day 30 over Day 15, Day 60 over Day15, and Day 60 over Day 30).

Table S1. Taxonomic comparison of select subjects (*n* =11) vs. the remainder of the subject population (*n* = 18): Day 15 vs. 30.

Taxonomy	Log2(Fold-change)	*P* value	Cohen's *D*	Functional Connotations
c__Erysipelotrichia	-1.134	0.073	0.829	Found to be enriched in colorectal cancer [1]
o__Erysipelotrichales	-1.134	0.073	0.829	Found to be enriched in colorectal cancer [1]
f__*Erysipelotrichaceae*	-1.134	0.073	0.829	Found to be enriched in colorectal cancer [1]
s__*Ruminococcus_callidus*	4.038	0.075	0.845	Increases Butyrate production [2]
g__*Ruminococcus*	1.220	0.085	0.788	Increases Butyrate production [2]

[1]Taxonomic hierarchies are designated as c (class), o (order), f (family), g (genus) or s (species).

Table S2. KEGG pathways altered when comparing Day 15 vs. 30 of select subjects (*n* = 11) vs. the remainder of the study group (*n* = 18).

Pathway	Log2(Fold-change)	*P* value	Cohen's *D*

ABC Transporters, Prokaryotic Type; ABC-2 type and other transporters; Heme transporter [MD:M00259]	-1.3406	0.0449	0.9626
Phosphotransferase System (PTS); Enzyme II [TC:4.A]; Glucose-specific II component [MD:M00265]	-0.7534	0.0526	0.8796
Prokaryotic Type; Helix-turn-helix; Rrf2 family	-0.7219	0.0695	0.8204

Table S3. Enzyme modulation observed when comparing select subjects (n = 11) with the remainder of the subject population (n = 18): Day 15 vs. 30.

Enzymes	Log2(Fold-change)	***P* value**	**Cohen's *D***
3.6.1.41 apaH; *bis*(5'-nucleosyl)-tetraphosphatase (symmetrical)	-1.019	0.025	1.053
2.4.1.129 mrcA; penicillin-binding protein 1A	0.274	0.037	0.954
3.4.16.4 mrcA; penicillin-binding protein 1A	0.274	0.037	0.954
2.7.11.33 ppsR; [pyruvate, water dikinase]-phosphate phosphotransferase / [pyruvate, water dikinase] kinase	-0.959	0.038	0.961
2.7.4.28 ppsR; [pyruvate, water dikinase]-phosphate phosphotransferase / [pyruvate, water dikinase] kinase	-0.959	0.038	0.961
2.4.2.9 pyrR; pyrimidine operon attenuation protein / uracil phosphoribosyltransferase	1.856	0.054	0.919
2.3.3.13 leuA, IMS; 2-isopropylmalate synthase	0.183	0.058	0.872
3.6.3.20 ugpC; *sn*-glycerol 3-phosphate transport system ATP-binding protein	-0.726	0.058	0.885
2.10.1.1 moeA; molybdopterin molybdotransferase	-0.600	0.061	0.846

2.8.1.12 MOCS2B, moaE; molybdopterin synthase catalytic subunit	-1.482	0.066	0.870
1.13.11.2 catE; catechol 2,3-dioxygenase	2.887	0.067	0.872
7.1.1.2 ndhI; NAD(P)H-quinone oxidoreductase subunit I	-1.078	0.069	0.843
3.4.23.51 hycI; hydrogenase 3 maturation protease	-1.372	0.071	0.854

Table S4. Taxonomic comparison of select subjects (*n* =11) vs. the remainder of the subject population (*n* = 18): Day 30 vs. 60.

Taxonomy	Log2 (Fold-change)	*P* value	Cohen's *D*	Functional Connotations
c__Coriobacteriia	1.728	0.026	1.086	Bile acid metabolism [3]
f__*Coriobacteriaceae*	1.889	0.031	1.049	Bile acid metabolism [3]
g__*Collinsella*	1.859	0.033	1.031	*Collinsella* has been linked to pro-inflammatory dysbiosis in type 2 diabetes and with circulating insulin suggestive of a mechanism for promotion of NAFLD pathology [4].
o__Coriobacteriales	1.842	0.034	1.029	Bile acid metabolism [3]
s__*Collinsella_aerofaciens*	1.795	0.035	1.018	*C. aerofaciens* is the major utilizer of lactose in the human colon. Several studies demonstrated that *Collinsella* and *Bifidobacterium* can modify the host bile acids to modulate the virulence and pathogenicity of enteric pathogens [4]
s__*Anaerostipes_hadrus*	1.649	0.058	0.897	Produces butyric acid [5]

[1]Taxonomic hierarchies are designated as c (class), o (order), f (family), g (genus) or s (species).

Table S5. Enzyme modulation observed when comparing select subjects (*n* = 11) with the remainder of the subject population (n = 18): Day 30 vs. 60.

Enzymes	Log2 (Fold-change)	*P* value	Cohen's *D*
5.1.3.9 nanE; *N*-Acylglucosamine-6-phosphate 2-epimerase	0.944	0.001	1.632
2.10.1.1 moeA; molybdopterin molybdotransferase	1.013	0.002	1.646
1.17.1.5 ndhF; nicotinate dehydrogenase FAD-subunit	0.978	0.004	1.434
1.17.1.10 fdhB; formate dehydrogenase ($NADP^+$) beta subunit	0.943	0.006	1.364
3.5.3.6 arcA; arginine deiminase	1.485	0.010	1.295
2.7.1.191 PTS-Man-EIIB, manX; PTS system, mannose-specific IIB component	1.452	0.010	1.293
3.1.3.102 ybjI; FMN hydrolase / 5-amino-6-(5-phospho-D-ribitylamino)uracil phosphatase	1.225	0.010	1.283
3.1.3.104 ybjI; FMN hydrolase / 5-amino-6-(5-phospho-D-ribitylamino)uracil phosphatase	1.225	0.010	1.283
4.1.1.31 ppc; phosphoenolpyruvate carboxylase	1.434	0.013	1.224
2.7.2.2 arcC; carbamate kinase	1.118	0.013	1.240
2.7.1.156 cobP, cobU; adenosylcobinamide kinase / adenosylcobinamide-phosphate guanylyltransferase	0.980	0.015	1.188
2.7.7.62 cobP, cobU; adenosylcobinamide kinase / adenosylcobinamide-phosphate guanylyltransferase	0.980	0.015	1.188

5.2.1.8 PPIA; peptidyl-prolyl *cis-trans* isomerase A (cyclophilin A)	1.619	0.016	1.209
4.6.1.17 moaC, CNX3; cyclic pyranopterin monophosphate synthase	1.055	0.016	1.197
2.5.1.129 ubiX, bsdB, PAD1; flavin prenyltransferase	0.999	0.016	1.150
1.17.1.9 fdoG, fdchF, fdwA; formate dehydrogenase major subunit	1.211	0.016	1.143
7.1.1.2 ndhI; NAD(P)H-quinone oxidoreductase subunit I	1.783	0.017	1.200
2.1.3.15 accD; acetyl-CoA carboxylase carboxyl transferase subunit beta	1.018	0.017	1.163
6.4.1.2 accD; acetyl-CoA carboxylase carboxyl transferase subunit beta	1.018	0.017	1.163
7.1.1.2 nuoI; NADH-quinone oxidoreductase subunit I	1.054	0.019	1.125
3.4.23.51 hycI; hydrogenase 3 maturation protease	2.642	0.019	1.188
3.6.1.7 acyP; acylphosphatase	1.288	0.019	1.161
3.2.1.85 E3.2.1.85, lacG; 6-phospho-beta-galactosidase	1.970	0.019	1.169
3.6.1.41 apaH; *bis*(5'-nucleosyl)-tetraphosphatase (symmetrical)	0.957	0.023	1.105
1.1.1.301 apdH, APDH; D-arabitol-phosphate dehydrogenase	1.076	0.023	1.120
3.2.1.86 E3.2.1.86B, bglA; 6-phospho-beta-glucosidase	1.042	0.025	1.087
2.7.7.85 disA; diadenylate cyclase	1.991	0.026	1.099
1.3.1.1 preT; dihydropyrimidine dehydrogenase (NAD+) subunit PreT	1.009	0.027	1.043
1.3.8.2 crtN; 4,4'-diapophytoene desaturase	2.237	0.029	1.061
5.4.99.21 rluF; 23S rRNA pseudouridine2604 synthase	1.121	0.037	0.995
2.7.1.200 PTS-Gat-EIIA, gatA, sgcA; PTS system, galactitol-specific IIA component	1.570	0.039	0.990

2.7.1.200 PTS-Gat-EIIB, gatB, sgcB; PTS system, galactitol-specific IIB component	2.253	0.039	1.009
3.6.3.30 afuC, fbpC; iron(III) transport system ATP-binding protein	0.964	0.039	0.970
1.1.1.1 yiaY; alcohol dehydrogenase	1.220	0.040	0.976
2.1.1.10 mmuM, BHMT2; homocysteine *S*-methyltransferase	1.048	0.040	0.975
1.17.1.5 ndhS; nicotinate dehydrogenase small FeS subunit	1.158	0.042	0.973
3.6.3.17 rbsA; ribose transport system ATP-binding protein	1.006	0.042	0.976
4.2.1.114 aksD; methanogen homoaconitase large subunit	1.282	0.042	0.973
5.4.99.59 fcf2; dTDP-fucopyranose mutase	-2.406	0.043	0.973
3.2.1.93 treC; trehalose-6-phosphate hydrolase	1.685	0.044	0.969
4.1.2.40 gatY-kbaY; tagatose 1,6-diphosphate aldolase GatY/KbaY	1.532	0.044	0.975
1.17.1.10 fdhA; formate dehydrogenase (NADP+) alpha subunit	2.272	0.044	0.973
3.6.3.36 tauB; taurine transport system ATP-binding protein	1.063	0.045	0.962
3.2.1.86 E3.2.1.86A, celF; 6-phospho-beta-glucosidase	1.857	0.045	0.974
3.6.3.29 modC; molybdate transport system ATP-binding protein	4.102	0.045	0.969
2.3.1.222 pduL; phosphate propanoyltransferase	1.229	0.046	0.956
2.7.7.76 mocA; molybdenum cofactor cytidylyltransferase	1.542	0.048	0.946

Table S6. KEGG pathways altered when comparing Day 30 vs. 60 of select subjects (n = 11) vs. the remainder of the study group (n = 18).

**Pathway**	**Log2(Fold-change)**	***P* value**	**Cohen's *D***
Prokaryotic Type; Helix-turn-helix; Rrf2 family	1.2016	0.0012	1.7058
2. Transferases; 2.10 Transferring molybdenum- or tungsten-containing groups; 2.10.1 Molybdenumtransferases or tungstentransferases with sulfide groups as acceptors	1.0202	0.0020	1.6439
ABC Transporters, Prokaryotic Type; Peptide and nickel transporters; Peptides/nickel transporter [MD:M00239]	1.0918	0.0043	1.4841
OmpR family; CusS-CusR	0.8147	0.0091	1.2592
Phosphotransferase System (PTS); Enzyme II [TC:4.A]; Mannose-specific II component [MD:M00276]	1.2367	0.0181	1.1455
3. Hydrolases; 3.5 Acting on carbon-nitrogen bonds, other than peptide bonds; 3.5.- Acting on carbon-nitrogen bonds, other than peptide bonds	1.6857	0.0199	1.1522
5. Isomerases; 5.-; 5.-.	1.5249	0.0223	1.1376
1. Oxidoreductases; 1.20 Acting on phosphorus or arsenic in donors; 1.20.4 With disulfide as acceptor	0.9183	0.0237	1.0807
Non-ion channels; Aquaglyceroporins or glycerol-uptake facilitators	0.8270	0.0239	1.0519
Serine Peptidases; Family S11: D-Ala-D-Ala carboxypeptidase A family	0.8015	0.0253	1.0484
Metallo Peptidases; Family M38: beta-aspartyl dipeptidase family	1.2947	0.0255	1.0991
Phosphotransferase System (PTS); Enzyme II [TC:4.A]; Trehalose-specific II component [MD:M00270]	1.2508	0.0293	1.0602
Aspartic Peptidases; Family A31: HybD endopeptidase family	1.0621	0.0401	0.9750
Metallo Peptidases; Family M13: neprilysin family	-0.8807	0.0475	0.9199

Table S7. Taxonomic comparison of select subjects (*n* =11) vs. the remainder of the subject population (*n* = 18): Day 15 vs 60

Enzymes	Log2 (Fold change)	*P* value	Cohen's *D*	Functional Connotations
g__*Butyricimonas*	-0.877	0.063	0.846	Related to immunodeficiency [6]
s__*Collinsella_aerofaciens*	1.225	0.112	0.728	C. aerofaciens is the major utilizer of lactose in the human colon. Several studies demonstrated that Collinsella and Bifidobacterium can modify the host bile acids to modulate the virulence and pathogenicity of enteric pathogens [4]
g__*Ruminococcus*	1.100	0.116	0.707	Increases Butyrate production [2]
g__*Mitsuokella*	-3.582	0.124	0.713	Butyrate producers [7]
c__Coriobacteriia	0.911	0.132	0.682	Bile acid metabolism [3]
o__Bacillales	1.321	0.135	0.677	Broad range biological activities [8]
s__*Bifidobacterium_bifidum*	2.128	0.140	0.675	Probiotic function [9]

[1]Taxonomic hierarchies are designated as c (class), o (order), f (family), g (genus) or s (species).

Table S8. Enzyme modulation observed when comparing select subjects (*n* = 11) with the remainder of the subject population (n = 18): Day 15 vs. 60.

Enzymes	Log2 (Fold-change)	*P* value	Cohen's *D*
3.4.23.51 hycI; hydrogenase 3 maturation protease	1.269	0.007	1.323
2.8.3.21 caiB; L-carnitine CoA-transferase	1.985	0.011	1.299

2.1.3.6 ptcA; putrescine carbamoyltransferase	1.420	0.013	1.189
2.4.2.52 citG; triphosphoribosyl-dephospho-CoA synthase	1.423	0.013	1.233
1.2.1.58 padG; phenylglyoxylate dehydrogenase alpha subunit	1.037	0.013	1.175
3.1.4.52 yahA; c-di-GMP-specific phosphodiesterase	1.230	0.024	1.064
2.7.7.7 dnaE2; error-prone DNA polymerase	3.389	0.024	1.105
2.4.2.18 trpGD; anthranilate synthase/phosphoribosyltransferase	0.911	0.027	1.028
4.1.3.27 trpGD; anthranilate synthase/phosphoribosyltransferase	0.911	0.027	1.028
1.3.1.44 fabV, ter; enoyl-[acyl-carrier protein] reductase / *trans*-2-enoyl-CoA reductase (NAD+)	1.496	0.030	1.051
1.3.1.9 fabV, ter; enoyl-[acyl-carrier protein] reductase / *trans*-2-enoyl-CoA reductase (NAD+)	1.496	0.030	1.051
1.17.1.5 ndhF; nicotinate dehydrogenase FAD-subunit	0.981	0.031	1.028
1.1.1.14 SORD, gutB; L-iditol 2-dehydrogenase	1.198	0.032	1.032
1.1.1.215 ghrB; glyoxylate/hydroxypyruvate/2-ketogluconate reductase	1.815	0.033	1.033
1.1.1.79 ghrB; glyoxylate/hydroxypyruvate/2-ketogluconate reductase	1.815	0.033	1.033
1.1.1.81 ghrB; glyoxylate/hydroxypyruvate/2-ketogluconate reductase	1.815	0.033	1.033
4.2.1.113 menC; *O*-succinylbenzoate synthase	1.536	0.035	1.000
5.1.3.22 ulaE, sgaU, sgbU; L-ribulose-5-phosphate 3-epimerase	1.602	0.039	0.983
2.7.7.39 tagD; glycerol-3-phosphate cytidylyltransferase	1.425	0.039	0.980
4.1.2.40 gatY-kbaY; tagatose 1,6-diphosphate aldolase GatY/KbaY	1.585	0.042	0.986
4.1.2.29 iolJ; 6-phospho-5-dehydro-2-deoxy-D-gluconate aldolase	1.825	0.043	0.977
1.3.8.13 caiA; crotonobetainyl-CoA dehydrogenase	1.235	0.044	0.938
2.1.1.307 elmMIII; 8-demethyl-8-(2,3-dimethoxy-alpha-L-rhamnosyl)tetracenomycin-C 4'-*O*-methyltransferase	1.529	0.044	0.976
2.7.8.12 tagF; CDP-glycerol glycerophosphotransferase	1.503	0.046	0.959
5.4.99.4 mgm; 2-methyleneglutarate mutase	-2.848	0.046	0.942

3.2.1.86 E3.2.1.86A, celF; 6-phospho-beta-glucosidase	1.234	0.046	0.954
1.6.5.2 qorB; NAD(P)H dehydrogenase (quinone)	1.227	0.047	0.913

Table S9. KEGG pathways altered when comparing Day 15 vs. 60 of select subjects (*n* = 11) vs. the remainder of the study group (*n* = 18).

Pathway	Log2(Fold-change)	*P* value	Cohen's *D*
Major Facilitator Superfamily (MFS); Organic acid transporters; Metabolite:H^+ symporter (MHS) family [TC:2.A.1.6]	1.7305	0.0433	0.9697
ABC Transporters, Prokaryotic Type; Monosaccharide transporters; D-Xylose transporter [MD:M00215]	1.2287	0.0574	0.9035
Nonribosomal peptide synthetase (NRPS); Linear NRPS; Surfactin family lipopeptide synthetase	1.7921	0.0581	0.9030
3. Hydrolases; 3.8 Acting on halide bonds; 3.8.1 In carbon-halide compounds	0.8357	0.0666	0.8508
ABC Transporters, Prokaryotic Type; Phosphate and amino acid transporters; Histidine transporter [MD:M00226]	1.2902	0.0673	0.8587
1. Oxidoreductases; 1.5 Acting on the CH-NH group of donors; 1.5.3 With oxygen as acceptor	2.2701	0.0729	0.8435
Phosphotransferase System (PTS); Enzyme II [TC:4.A]; Ascorbate-specific II component [MD:M00283]	0.9667	0.0731	0.8176
Prokaryotic Type; Helix-turn-helix; LuxR family	0.8264	0.0796	0.8123
ABC Transporters, Prokaryotic Type; Monosaccharide transporters; Multiple sugar transporter [MD:M00216]	1.3724	0.0894	0.7958

30 over 15 days

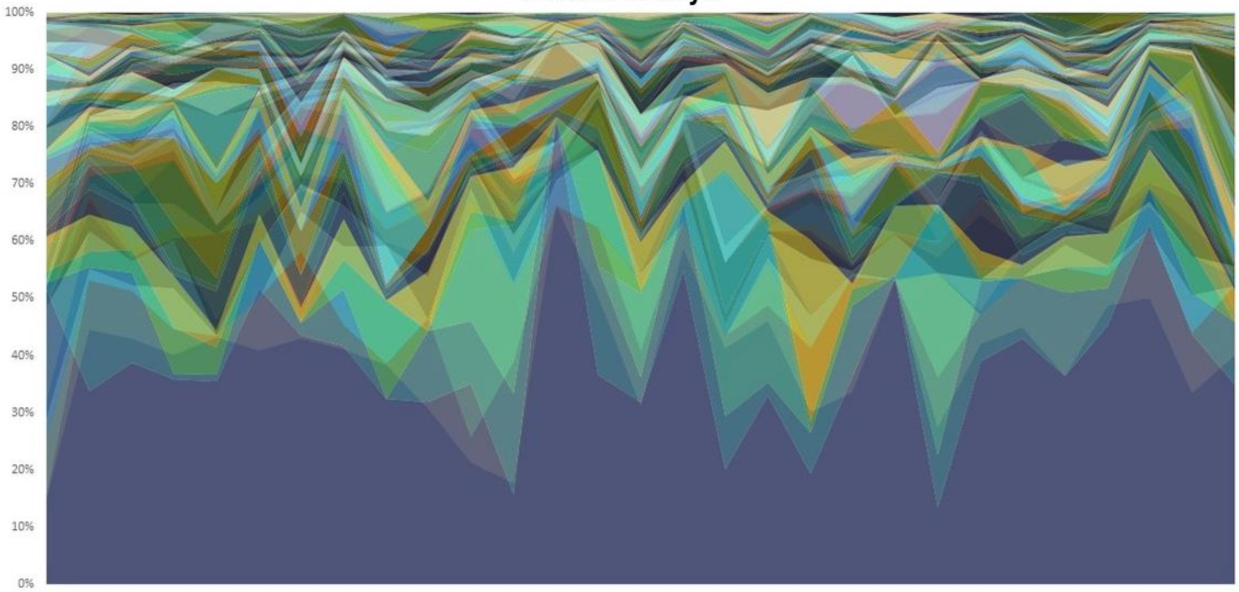

60 over 15 days

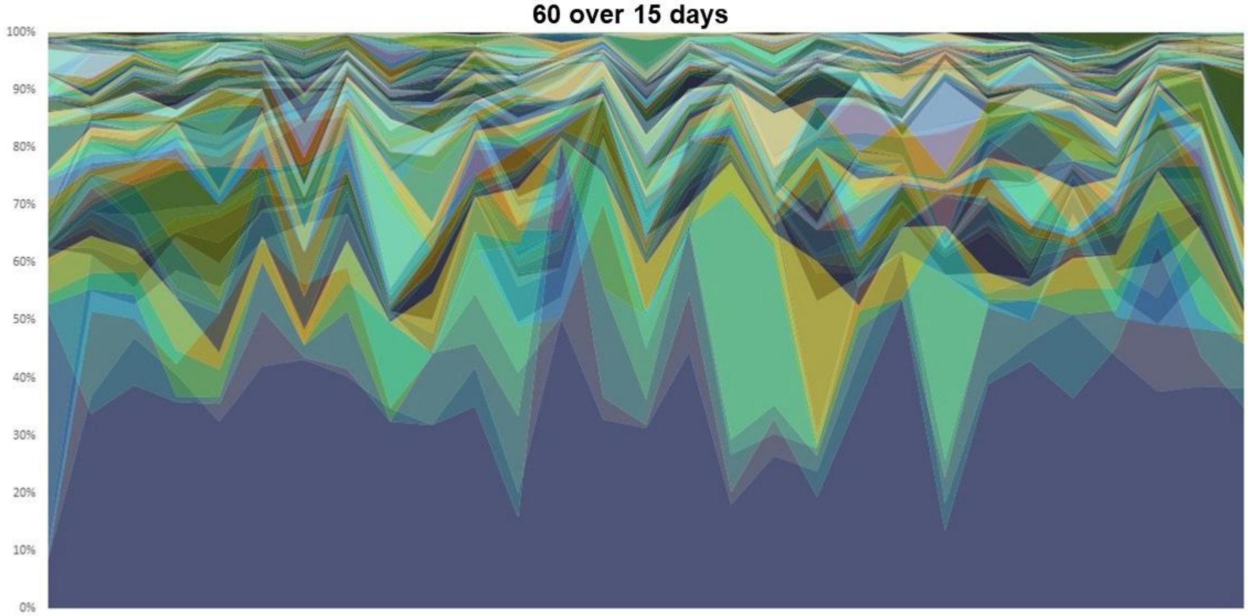

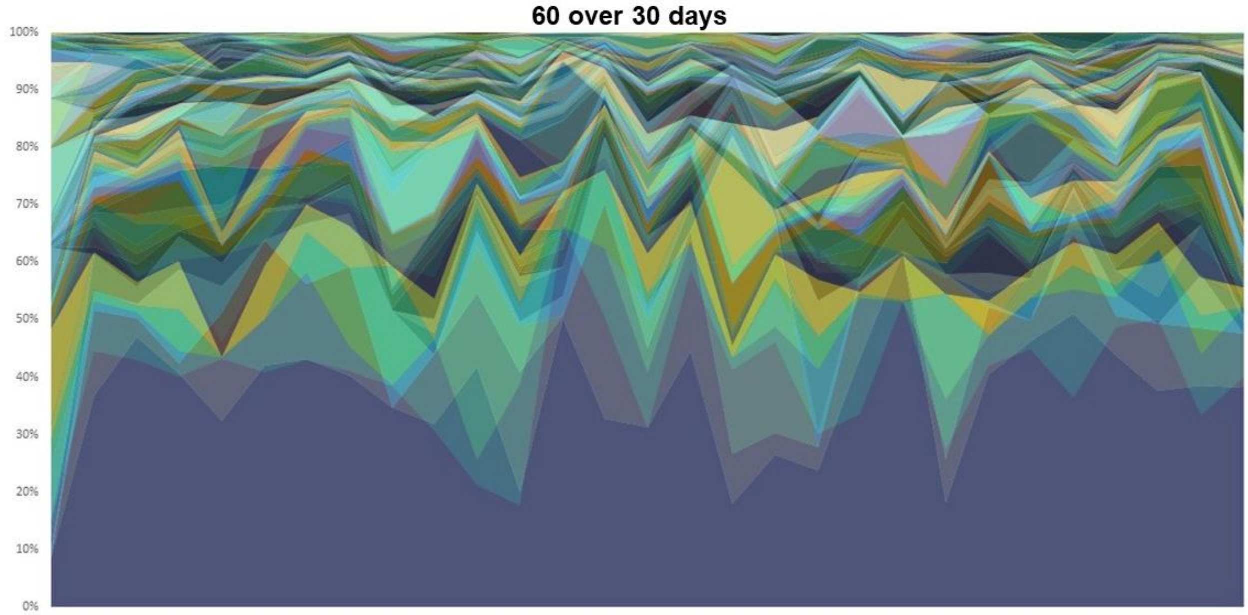

Figure S1. Area chart representing diversity of species. Overlays shown for Day 30 over Day 15, Day 60 over Day15, and Day 60 over Day 30.

References cited in Supplement 3.

[1] N.O. Kaakoush, Insights into the Role of Erysipelotrichaceae in the Human Host, Front Cell Infect Microbiol. 5 (2015) 84. https://doi.org/10.3389/fcimb.2015.00084.

[2] H. Nagao-Kitamoto, N. Kamada, Host-microbial Cross-talk in Inflammatory Bowel Disease, Immune Netw. 17 (2017) 1–12. https://doi.org/10.4110/in.2017.17.1.1.

[3] S.L. Cheng, X. Li, H.-J. Lehmler, B. Phillips, D. Shen, J.Y. Cui, Gut Microbiota Modulates Interactions Between Polychlorinated Biphenyls and Bile Acid Homeostasis, Toxicol Sci. 166 (2018) 269–287. https://doi.org/10.1093/toxsci/kfy208.

[4] J. Chen, K. Wright, J.M. Davis, P. Jeraldo, E.V. Marietta, J. Murray, H. Nelson, E.L. Matteson, V. Taneja, An Expansion of Rare Lineage Intestinal Microbes Characterizes Rheumatoid Arthritis, Genome Medicine. 8 (2016) 43. https://doi.org/10.1186/s13073-016-0299-7.

[5] R. Kant, P. Rasinkangas, R. Satokari, T.E. Pietilä, A. Palva, Genome Sequence of the Butyrate-Producing Anaerobic Bacterium *Anaerostipes hadrus* PEL 85, Genome Announc. 3 (2015) e00224-15. https://doi.org/10.1128/genomeA.00224-15.

[6] N. Ulger Toprak, T. Bozan, Y. Birkan, S. Isbir, G. Soyletir, *Butyricimonas virosa*: The First Clinical Case of Bacteraemia, New Microbes New Infect. 4 (2015) 7–8. https://doi.org/10.1016/j.nmni.2014.12.004.

[7] S. Kalyana Chakravarthy, R. Jayasudha, K. Ranjith, A. Dutta, N.K. Pinna, S.S. Mande, S. Sharma, P. Garg, S.I. Murthy, S. Shivaji, Alterations in the Gut Bacterial Microbiome in Fungal Keratitis Patients, PLoS One. 13 (2018) e0199640. https://doi.org/10.1371/journal.pone.0199640.

[8] O.N. Ilinskaya, V.V. Ulyanova, D.R. Yarullina, I.G. Gataullin, Secretome of Intestinal Bacilli: A Natural Guard Against Pathologies, Frontiers in Microbiology. 8 (2017). https://www.frontiersin.org/articles/10.3389/fmicb.2017.01666 (accessed September 17, 2022).

[9] F. Turroni, S. Duranti, C. Milani, G.A. Lugli, D. van Sinderen, M. Ventura, *Bifidobacterium bifidum:* A Key Member of the Early Human Gut Microbiota, Microorganisms. 7 (2019) 544. https://doi.org/10.3390/microorganisms7110544.

Supplement 4. Metabolomic analysis of select individuals.

Plasma metabolites determined by GC-MS in plasma (Panel A) and urine (Panel B) with the group of 11 select individuals (M1) and the remaining 18 subjects (M2). No statistically significant differences (nonparametric Mann-Whitney tests) were observed other than a slight decrease in *myo*-inositol in plasma (p = 0.03).

A

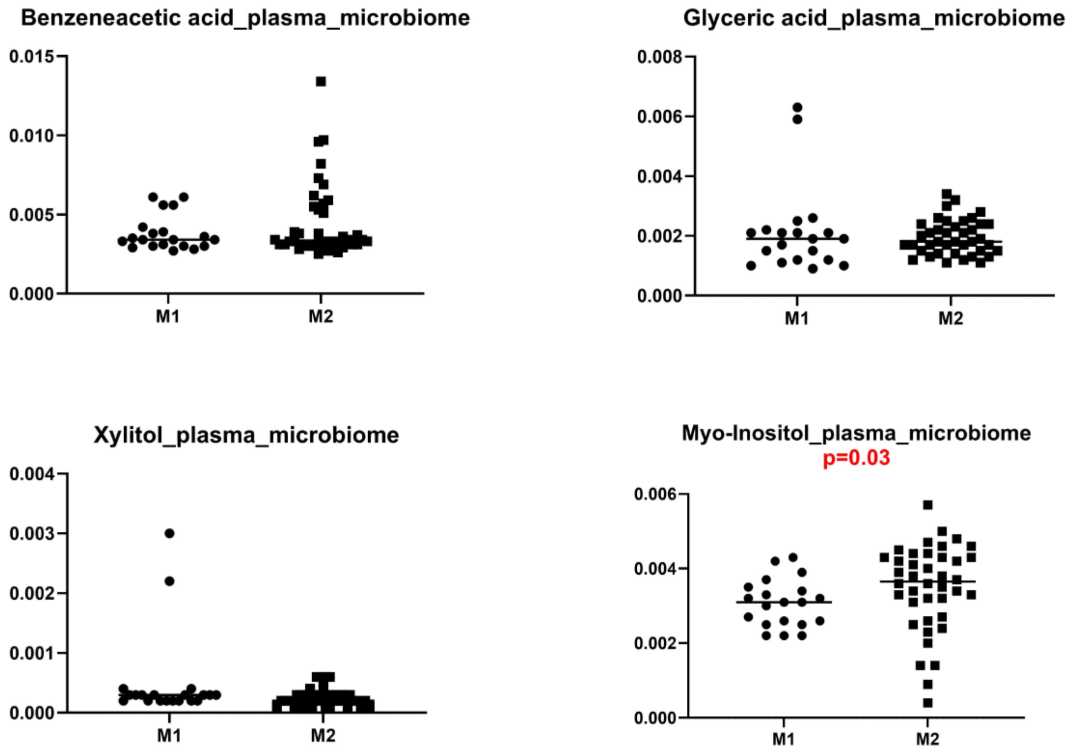

B

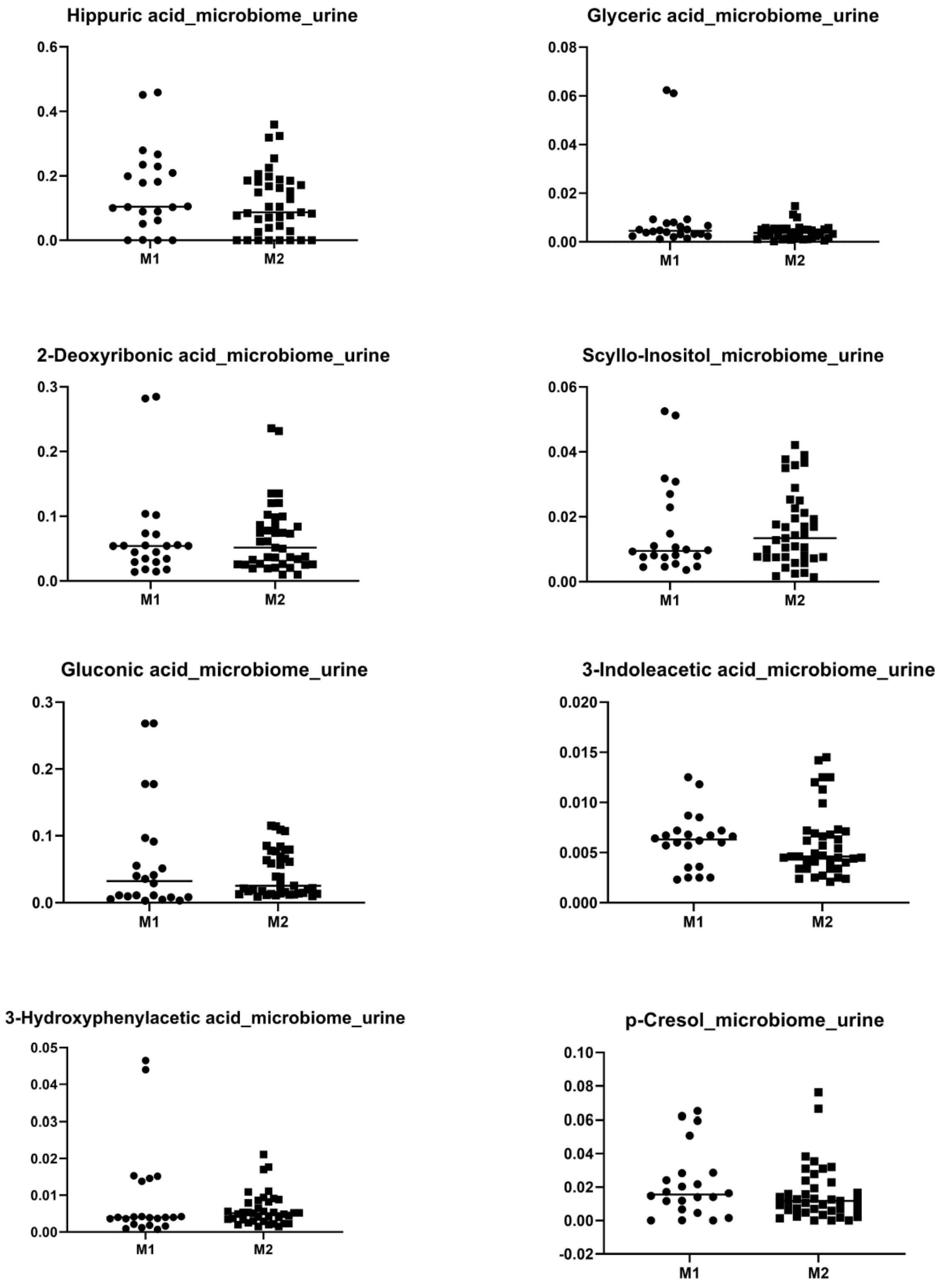

Hippuric acid_microbiome_urine
Glyceric acid_microbiome_urine
2-Deoxyribonic acid_microbiome_urine
Scyllo-Inositol_microbiome_urine
Gluconic acid_microbiome_urine
3-Indoleacetic acid_microbiome_urine
3-Hydroxyphenylacetic acid_microbiome_urine
p-Cresol_microbiome_urine
M1
M2

Supplement 5. Comparison of select taxa with the subject population: Day 15 vs. 30.

Taxonomy	Log2(Fold-change)	*P* value	Cohen's *D*
s__*Parabacteroides_johnsonii*[1]	-1.467	0.391	0.293
s__*Parabacteroides_distasonis*	1.088	0.276	0.292
g__*Parabacteroides*	0.548	0.251	0.309
s__*Parabacteroides_merdae*	0.175	0.714	0.107
f__*Clostridiaceae*	0.446	0.169	0.253
c__Clostridia	-0.009	0.955	0.086
o__Clostridiales	-0.009	0.955	0.086
f__*Clostridiales_Family_XIII._Incertae_Sedis*	0.132	0.739	0.104
g__*Klebsiella*	-1.968	0.390	0.226
s__*Klebsiella_pneumoniae*	-0.210	0.893	0.028
s__*[Clostridium]_leptum*	2.316	0.111	0.364
s__*[Clostridium]_bolteae*	1.109	0.241	0.365
g__*Clostridium*	0.548	0.116	0.327
s__*Clostridium*_sp._L2-50	2.533	0.281	0.113
s__*Clostridium*_sp._AT4	0.757	0.296	0.276
s__*[Clostridium] clostridioforme*	0.697	0.081	0.492

[1]Taxonomic hierarchies are designated as c (class), o (order), f (family), g (genus) or s (species).

Supplement 6. Inclusion/Exclusion Criteria

2.1.1 Inclusion Criteria

A subject was considered eligible for participation in the study if all of the following inclusion criteria were satisfied prior to randomization:

1. Was a healthy male or female (confirmed by medical history);
2. Was between the ages of 19 - 55 years of age;
3. Was a non-smoker;
4. Maintained a healthy weight;
5. Consumed little to no alcoholic beverages, or in moderation when consumed;
6. Had not consumed recreational drugs for one week prior;
7. Was willing to abstain from chronic acetaminophen, NSAID and COX-2 inhibitor use for duration of study;
8. Agreed not to participate in any clinical at Day 1 through study completion;
9. In the case of a female of childbearing potential was using two acceptable forms of birth control since last menses (oral/implant/injectable/transdermal contraceptives, intra vaginal ring, intrauterine device (IUD), condom, diaphragm, spermicidal agent, abstinence, partner's vasectomy, tubal ligation). Abstinence or vasectomies were acceptable if the female subject agreed to implement two of the other acceptable methods of birth control if her lifestyle/partner changed;
10. In the case of a female of childbearing potential, had a negative urine pregnancy test (UPT) on Day 1 and was willing to submit to a UPT at the end of study (EOS);
11. In the case of a female of non-childbearing potential: had had a hysterectomy, surgical bilateral oophorectomy and/ or bilateral salpingectomy, or was postmenopausal (at least 1 year with no menses prior to enrollment);
12. Completed a medical screening procedure; and
13. Read, understood, and signed an informed consent.

2.1.2 Exclusion Criteria

A subject who had any of the following was excluded from the study:

1. Was using nasally inhaled/systemic/topical corticosteroids within 4 weeks prior to and/or during the study, or systemic/topical antihistamines 72 hours prior to and during the study;

2. Was using certain antifungal drugs, antihistamines (including diphenhydramine, or Benadryl), antibiotics (including "sulfa" drugs, quinolones and tetracyclines), oral diabetes drugs, sulfonylureas, diuretics, and tricyclic antidepressants. Some herbal supplements such as St. John's wort would have also made a person ineligible;

3. Was not willing to refrain from using acetaminophen (occasional use permitted, except within 48 hours of a study visit) or systemic/topical anti-inflammatory analgesics such as aspirin, Aleve, Motrin, Advil, Orudis,or Nuprin for 72 hours prior to and during the study;

4. Any of the following in the 4 weeks prior to start of study:

   a. Major surgery for any indication
   b. Was on cytotoxic chemotherapy for any indication (including methotrexate for arthritis)
   c. Hormonal therapy for cancer prevention (including tamoxifen). Note: treatment with finasteride/dutasteride for BPH did not render a participant ineligible
   d. Topical medications for treatment at the skin site being evaluated (retin A, Accutane, PUVA, 5-FU)
   e. Was taking medication known to cause phototoxic reactions (e.g., tetracyclines, thiazides, nonsteroidal anti-inflammatory drugs [NSAIDS])
   f. Was using medication which, in the opinion of the Investigator, would have interfered with the study results (e.g., anti-inflammatory medications, antipsychotics, anticonvulsants with potential pain relief effects, immunomodulatory medications);

10. Had a known sensitivity or allergy to constituents of the materials being evaluated;

11. Had nut allergies;

12. Was a female who was pregnant, planned to become pregnant during the study, or was breast feeding a child;

13. Had received treatment for any type of internal cancer within 5 years prior to study entry;

14. Had a history of or currently being treated for:

   a. Hepatitis;
   b. Diabetes;
   c. Solid organ or bone marrow transplant
   d. Keloid formation
   e. Chronic renal or hepatic disorder
   f. Significant bleeding disorder

15. OTHER

   a. Had any condition that might have compromised study results;
   b. Was currently participating in any clinical testing;
   c. Had received any investigational drug(s) within 28 days from Day 1.

16. Uncontrolled concurrent illness including ongoing or active infection, symptomatic congestive heart failure, unstable angina pectoris, uncontrolled symptomatic cardiac arrhythmia, psychiatric illness/social situations that limited compliance with study requirements or other underlying serious medical condition which, in the investigator's opinion precluded study participation.

Supplement 7. Subject Responsibilities:

- Products to be excluded during the study period including those with alpha-hydroxy acids (AHAs like glycolic acid), beta-hydroxy acids (BHAs like salicylic acid); tretinoins (like Retin A); and benzoyl peroxide.
- Avoid celery, dill, fennel, figs, lime, and parsley.
- Avoid citrus, green tea, almonds, red fruits and vegetables, turmeric, olive oil.

Do not to consume the following foods during the study:

- Artichokes
- Berries (blueberries, blackberries, grapes, raspberries, strawberries, goji berries, etc.)
- Cocoa
- Dark chocolate
- Pomegranate
- Red wine

Limit the following foods during the study:

- Coffee/Tea – 1X per day
- Beans/Legumes – 2X per week
- Soy foods (tofu, soy milk, miso, tempeh) 2X per week

Do not consume the following supplements during the study:

- Multivitamin
- Alpha lipoic acid
- B vitamins
- Coenzyme Q10

- Elderberry
- Ellagic acid
- Fish oil
- Flaxseed or flaxseed oil
- Grapeseed extract
- Green tea
- Lycopene
- Niacinamide
- Quercetin
- Resveratrol
- Selenium
- Turmeric
- Vitamin C
- Vitamin E
- Vitamin K

Supplement 8. Dosing Protocol for Grape Powder

Important Information:

- Grape powder packets should be stored in a freezer until use.
- Hygroscopic material: protect from water and humidity until reconstituted.
- Drink within 30 minutes of reconstitution.
- Re-stir the grape powder and water just prior to drinking. Please note that the powder doesn't dissolve but creates a suspension.

Purpose: To disperse 36 g of grape powder in 180 ml (6 fl. oz.) of water.

Equipment:

- Clean glass or cup
- Volumetric measuring device
- Filtered or tap water (for reconstitution and rinse)

Procedure:

Step	Instructions
1.	Add approximately 180 ml. (6 fl. oz) of water to cup
2.	Open 36 g packet of grape powder and pour into water in cup.
3.	Stir well, about 30 seconds. If there is any clumping of the powder, break up the clumps by pressing them with the spoon against the glass.
4.	Visually confirm that no un-wetted powder remains. Continue stirring if needed.
5.	Drink within 30 minutes of reconstitution.
6.	Time of Dosing ____________
7.	Rinse container with at least 30 mL (I fl. oz.) of water and drink to ensure getting all of the grape powder.
